# Single-cell transcriptomics highlights immunological dysregulations of monocytes in the pathobiology of COPD

**DOI:** 10.1186/s12931-022-02293-2

**Published:** 2022-12-20

**Authors:** Qiqing Huang, Yuanyuan Wang, Lili Zhang, Wei Qian, Shaoran Shen, Jingshen Wang, Shuangshuang Wu, Wei Xu, Bo Chen, Mingyan Lin, Jianqing Wu

**Affiliations:** 1 Key Laboratory of Geriatrics of Jiangsu Province, Department of Geriatrics, The First Affiliated Hospital of Nanjing Medical University, 300 Guangzhou Road, Nanjing, 210029 Jiangsu China; 2 State Key Laboratory of Reproductive Medicine, Nanjing Medical University, Nanjing, 211166 Jiangsu China; 3 Department of Neurobiology, School of Basic Medical Sciences, Nanjing Medical University, 101 Longmian Avenue, Nanjing, 211166 Jiangsu China

**Keywords:** COPD, Aging, Smoking, Single-cell RNA sequencing, Monocytes

## Abstract

**Background:**

Chronic obstructive pulmonary disease (COPD) is a common respiratory disease, whose pathogenetic complexity was strongly associated with aging/smoking and poorly understood.

**Methods:**

Here we performed single-cell RNA sequencing (scRNA-seq) analysis of 66,610 cells from COPD and age-stratified control lung tissues of donors with different smoking histories to prioritize cell types most perturbed in COPD lungs in aging/smoking dependent or independent manner. By performing an array of advanced bioinformatic analyses, such as gene set enrichment analysis, trajectory analysis, cell–cell interactions analysis, regulatory potential analysis, weighted correlation network analysis, functional interaction analysis, and gene set variation analysis, we integrated cell-type-level alterations into a system-level malfunction and provided a more clarified COPD pathological model containing specific mechanisms by which aging and smoking facilitate COPD development. Finally, we integrated the publicly available scRNA-seq data of 9 individuals, resulting in a total of 110,931 cells, and replicated the analyses to enhance the credibility of our findings.

**Results:**

Our study pointed to enrichment of COPD molecular alteration in monocytes, which further induced a previously unrecognized pro-inflammatory effect on alveolar epithelial cells. In addition, aged monocytes and club cells facilitated COPD development via maintaining an autoimmune airway niche. Unexpectedly, macrophages, whose defect to resolve inflammation was long-recognized in COPD pathogenesis, primarily induced an imbalance of sphingolipids rheostat in a smoking-dependent way. These findings were validated in a meta-analysis including other public single-cell transcriptomic data.

**Conclusions:**

In sum, our study provided a clarified view of COPD pathogenesis and demonstrated the potential of targeting monocytes in COPD diagnosis and treatment.

**Supplementary Information:**

The online version contains supplementary material available at 10.1186/s12931-022-02293-2.

## Background

As a leading cause of chronic morbidity and mortality throughout the world, COPD results from a complex interaction of genetic predisposition and environmental exposures [1], which involves a number of pathological processes, including oxidative stress [2], protease-antiprotease imbalance [3], inflammatory cells and mediators [4] as well as recently reported autoimmunity [5], metabolic reprogramming [6], perturbations in the pulmonary microbiome [7], and accelerated cellular senescence [8]. On the other hand, the pathogenesis of COPD usually manifests clinically in individuals at an advanced age after years of cigarette smoking, lending credence to the notion that aging and smoking together with their biological consequences are important mechanisms in disease pathogenesis [9]. In spite of all the aging hallmarks [10] and inflammaging [11, 12] observed in COPD patients, the implications of aging in COPD pathogenesis have not been fully understood. The same is true of smoking. Cigarette-smoke-exposed lung tissues manifest complicatedly in terms of disease types, pathogenesis sites, and cellular composition [13]. New insights such as iron dyshomeostasis [14], lipidomic remodelling [15], and changes in haem metabolism [16] enriched our mechanistic understanding of smoking in COPD development while also complicating the issue. Therefore, one key to the conundrum of pathological complexity is to disentangle COPD heterogeneity while discerning the roles of these two well-recognized risk factors for facilitating COPD. Unfortunately, few studies have actually addressed this challenge, nor have they fully clarified the central pathological features and mechanisms of COPD.

Recent studies have adopted single-cell RNA sequencing (scRNA-seq) to explore cell-type-specific mechanisms of COPD [17–21]. Herein, by taking advantage of this single-cell resolution technology along with well-designed analytic strategy, we sought to assess COPD-associated alteration independent of smoking and aging (defined as “COPD core” pathology), and clarify the facilitative mechanisms of aging and smoking. To this end, we analysed the single-cell transcriptomic data of more than 65,000 cells in lung tissues from COPD patients, and healthy elderly as well as young donors, with different smoking histories. To enhance the credibility of our findings, we replicated the analyses by including the scRNA-seq data of 9 individuals from a recent publication [22], resulting in a total of 110,931 cells. Collectively, our study prioritized cell types under COPD, aging, and smoking conditions independently; depicted system-level malfunctions under each condition based on cell-type-level alterations; and further established a clarified “COPD core” pathological model containing specific mechanisms that mediate the contributions of aging and smoking to COPD development. By sorting out the heterogeneity of both the disease and the major risk factors, we provide simpler and clearer insights into COPD pathogenesis involving aging and smoking, underscoring the potential of previously unappreciated monocytes as an early warning and therapeutic target for COPD.

## Methods

### Human lung tissue samples and ethics statement

This study was approved by the Ethics Committee at the First Affiliated Hospital of Nanjing Medical University (IRB-GL1-AF08). We complied with all relevant ethical regulations and written informed consent was obtained from each patient prior to surgery. Only patients with untreated, primary, non-metastatic lung tumors that underwent lung lobe resection with curative intent were included and were divided into three groups according to lung function and age: COPD (according to the GOLD guidelines) group (2 active smokers and 1 never-smoker, 62 ± 11.53), normal elderly (≥ 65 years) group (2 active smokers and 1 never-smoker, 73 ± 2.00), and normal young (≤ 40 years) group (3 never-smokers, 30 ± 4.36). No patients had a history of asthma or renal dysfunction. The clinical and histological data of the patients are shown in Supplementary Data1 and Additional file 2: Fig. S1A. During surgery, all samples were obtained from resected lobes more than 5 cm away from the tumor border to get rid of the influence of cancer.

### Single-cell RNA sequencing data analysis

The BD Rhapsody analysis pipeline was used to process sequencing data and the reference genome was GENCODE v29. Expression matrix was processed with the Seurat (version 3.1.5). The cells were removed that had either fewer than 301 expressed genes or over 30% unique molecular identifiers (UMIs) originating from mitochondria. UMI counts were normalized and were transformed to the log-transformed. Integration of nine dataset was performed to correct batch effect. Visualization of transcriptomic profiles were conducted by uniform manifold approximation and projection (UMAP). The Louvain modularity optimization algorithm was applied to iteratively group cells together into clusters. Cell clusters were annotated to known biological cell types using canonical cell marker genes. Differential expression analysis was performed using the MAST (1.14.0). Cell types most relevant to one specific condition was ranked by the number of DEGs after taking the size of cells into account. Cell type prioritization analysis under three conditions (COPD, aging, and smoking) was done using Augur R package (version 1.0.0). Gene Ontology enrichment analysis and gene set enrichment analysis (GSEA) were performed using the clusterProfiler (version 3.14.3). Gene set variation analysis (GSVA) was conducted using GSVA R package (version 1.34.0). Enrichment of DEGs among COPD GWAS risk genes was performed using Fisher's exact test, with all genes detected > 10% of cells in each cluster used as the background. Co-expression network of alveolar type 2 cells was performed using weighted gene coexpression network analysis (WGCNA) (version 1.69). Developmental trajectory analysis was performed using the Slingshot (version 1.4.0) and genes dynamically expressed during cell differentiation was found by tradeSeq package (version 1.4.0). Cell–cell interactions (CCIs) analysis was conducted with the CellPhoneDB (version 2.0). Intercellular communication was inferred using iTALK (version 0.1.0) (Corpus ID: 91,802,336) and nichenetr R package (version 1.0.0). For a full list of methods with more details, see Additional file 1: Supplementary Methods.

## Results

### Cell-type-specific gene expression changes under different conditions

**Fig. 1:**
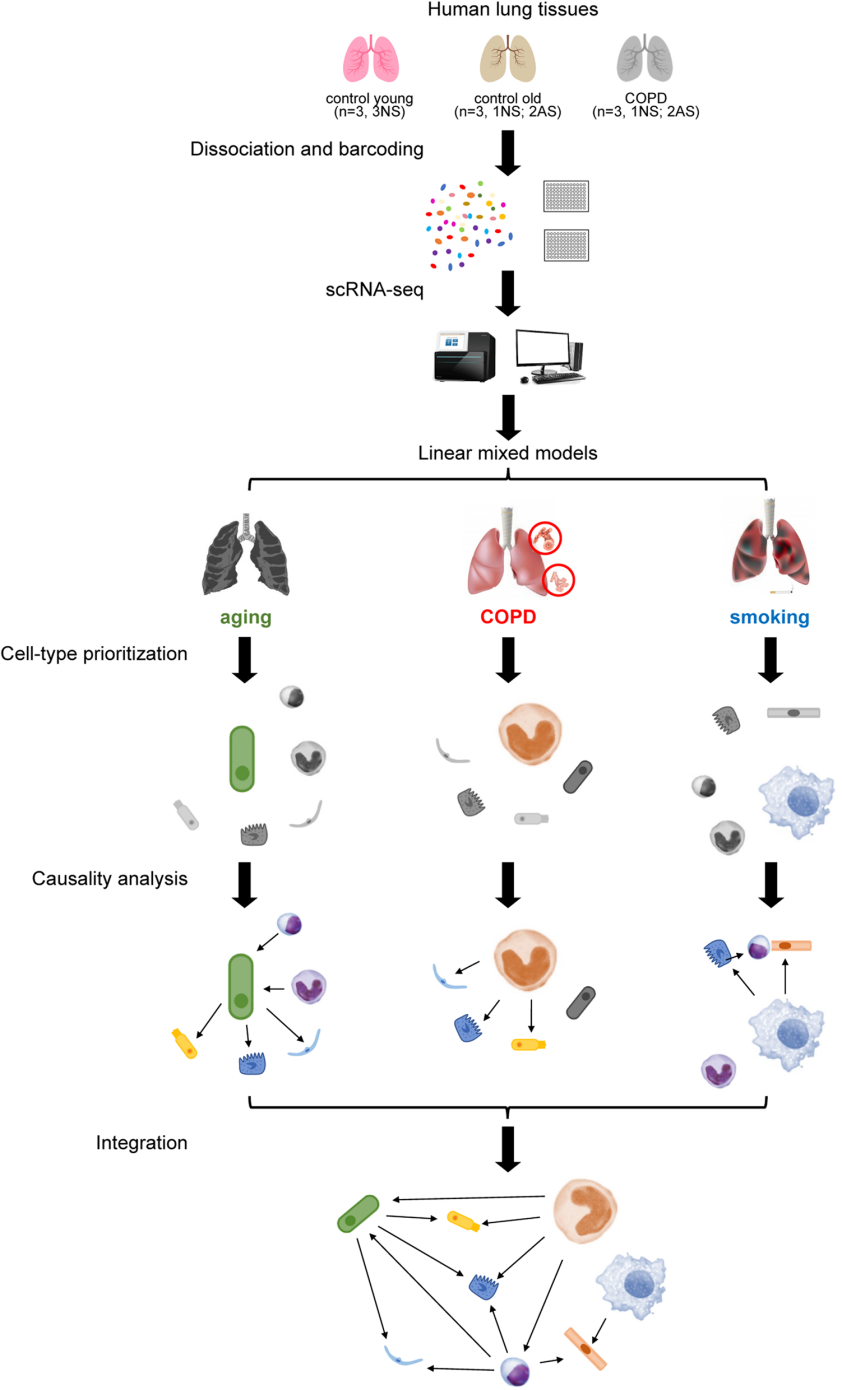
Schematics of scRNAs analyses. Lung tissue samples were from 3 COPD patients (COPD), 3 age-matched controls (control old), and 3 young individuals (control young), among which 4 were active smokers (AS) and 5 were never-smokers (NS). Upon dissociation and barcoding, scRNA-seq was carried out. Following data deconvolution with a linear mixed model, cell type prioritization analysis was performed under 3 different pathological conditions, including COPD, aging, and smoking, to identify the most affected cell types under each condition (the biggest colored cell type). By causality analysis between the prioritized cell types with other cell types, cell type level alterations were integrated into 3 condition-specific system-level malfunctions, of which the one under COPD condition was defined as “COPD core” pathology. Finally, a new comprehensive COPD pathological model taking aging and smoking into account was established by combining system-level malfunctions under all conditions

We performed scRNA-seq on lung tissue samples from 3 COPD patients (62 ± 11.53) and 6 controls with varied histories of smoking (4 active smokers and 5 never-smokers) (Fig. 1, Additional file 3: Dataset 1, and see Additional file 1: Supplementary Methods). Among controls, 3 were age-matched to COPD patients (72 ± 2.00), while the rest were from young individuals (30 ± 4.36). Hence, by deconvolving molecular complexity with a linear mixed model, we were able to characterize the molecular pathogenesis with the roles of aging and smoking assessed independently (see Additional file 1: Supplementary Methods). Data of scRNA-seq are notorious for false-positive and trivial findings as a result of the large amount of data. Therefore, to identify the most prominent alteration not only at the level of individual cell types but also in the whole system, we adopted the following analytic strategy. Starting with cell-type prioritization analysis, we first identified the most affected cell types under each condition, including COPD, aging, and smoking. Subsequently, we explored causality between the prioritized cell types and others with an array of advanced bioinformatic tools, such as CCIs analysis, integrating cell-type-level alterations into a system-level malfunction. The system-level malfunction occurring under the COPD condition was defined as the “COPD core” pathology. Ultimately, we combined findings under all conditions and established a new comprehensive COPD pathological model in which how aging and smoking facilitate COPD development was also clarified (Fig. 1).

**Fig. 2:**
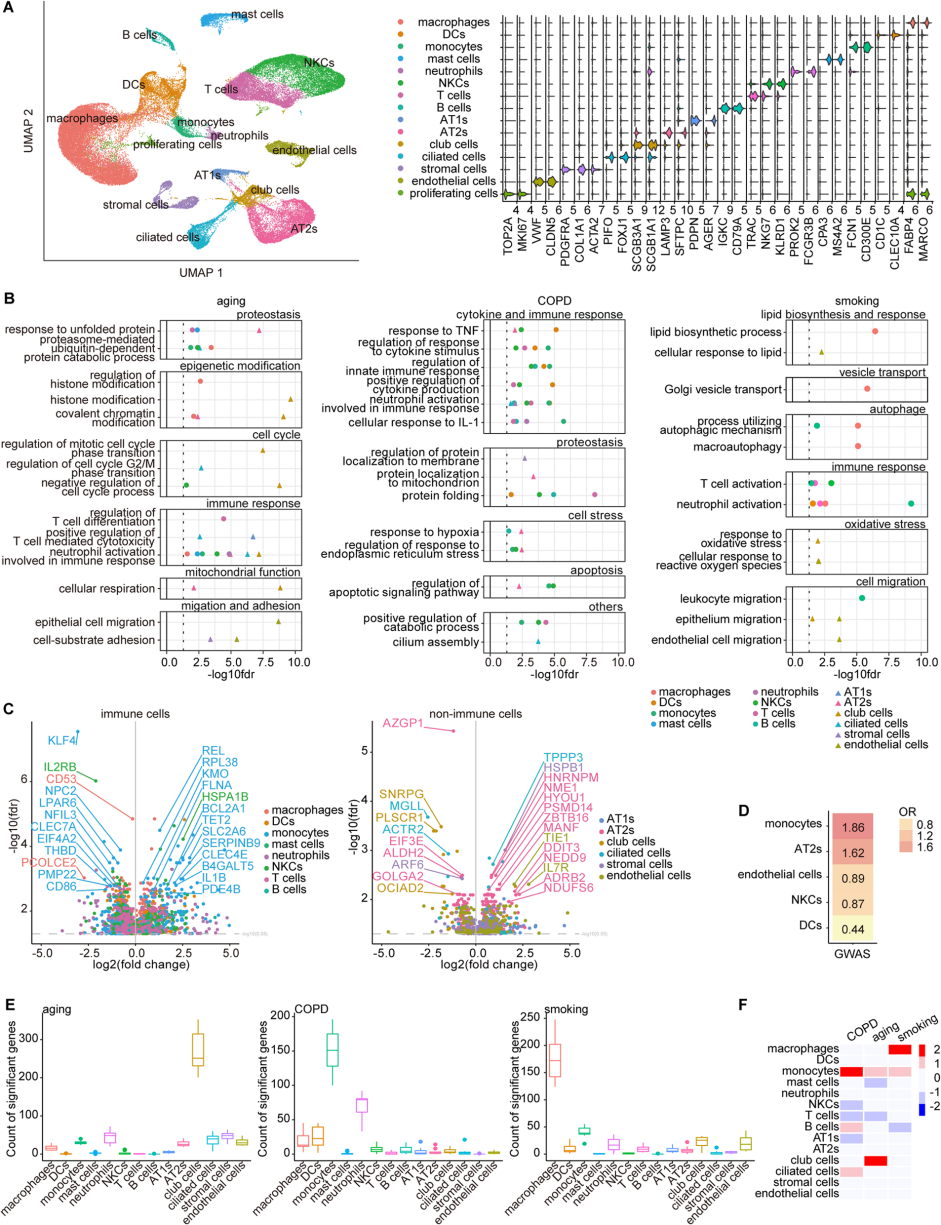
Cell-type-specific gene expression changes under different conditions. **A** UMAP plot of the 15 identified cell types is used to visualize the result of clustering of the 66,610 cells from all 9 lung tissue samples (left). Violin plots showing the expression of marker genes of each cell type (right). **B** Functional enrichment analysis showing biological processes in GO categories of cell-type specific DEGs associated with aging (left), COPD (middle), and smoking (left). Different colors represent different cell types, among which immune cells are displayed as triangles and non-immune cells as circles. **C** Volcano plots for COPD-associated DEGs expressed in immune cells (left) and non-immune cells (right). One dot represents a DEG. Different colors represent different cell types. **D** Heatmap showing the fisher’s exact test enrichment odds ratio of COPD GWAS risk genes in COPD-associated DEGs of each cell type. Only cell types with fisher’s exact test p-value < 0.05 are displayed. **E** Box plots showing the number of aging- (left), COPD- (middle), and smoking-associated DEGs (right) for all cell types normalized by the number of cells in downsampling analysis. **F** All the cell types except proliferating cells are prioritized according to the separability of perturbed and unperturbed cells within a high-dimensional space under the conditions of aging, COPD, and smoking in AUGUR analysis, respectively, which is visualized as heatmap. The color of the heatmap indicates high (red) or low (blue) cell type prioritization score

Upon quality control (Additional file 2: Fig. S1B–S1D) and data processing (see Additional file 1: Supplementary Methods), we classified cells into 15 cell types, including 14 known cell types (macrophages, dendritic cells (DCs), monocytes, mast cells, neutrophils, natural killer cells (NKCs), T cells, B cells, alveolar type 1 cells (AT1s), alveolar type 2 cells (AT2s), club cells, ciliated cells, stromal cells, and endothelial cells) and a distinct unknown cluster highly positive for cell proliferation markers named proliferating cells, which was also detected in a recently published scRNA-seq dataset of human lung tissues [23] (Fig. 2A, Additional file 2: Fig. S2A and S2B, and see Additional file 1: Supplementary Methods). In general, cell compositions were not biased towards any level of conditions (Additional file 2: Fig. S2C–S2E).

To independently assess molecular alterations associated with COPD, aging, and smoking in a cell-type-specific manner, we adopted a linear mixed model to identify differentially expressed genes (DEGs) under each condition (see Additional file 1: Supplementary Methods, and Additional file 4: Dataset 2). Expectedly, aging-associated DEGs were enriched with molecular hallmarks of aging [10], reflecting the general status of aged lungs from elderly subjects; the biological ontologies associated with smoking appeared to be the most heterogeneous, yet they still highlighted cigarette-smoking-induced cellular stress and changes in lipid metabolism and vesicle transport (Fig. 2B and Additional file 2: Fig. S2F). Notably, regulation of response to cytokine stimulus, cytokine production, and innate immune response were among the top dysregulated Gene Ontology (GO) terms associated with COPD, emphasizing the involvement of cytokines and the innate immune system in COPD pathogenesis. To further reveal genes responsible for COPD in particular, we showed that the top COPD-associated DEGs among immune cells were from monocytes, including nuclear factor-kappa B (NF-κB) signaling genes such as *REL*, *IL1B*, *KLF4* and *FLNA*; among non-immune cells, the top DEGs were from AT2s and club cells, including *AZGP1*, *SNRPG*, *HNRNPM*, *EIF3E*, *TIE1*, and *IL7R* (Fig. 2C). We also integrated genome-wide association study (GWAS) data [24] with COPD-associated genes to determine cell types contributing to COPD heritability and demonstrated that the GWAS COPD risk genes were mostly overrepresented among DEGs in monocytes, followed by AT2s and endothelial cells (Fig. 2D and see Additional file 1: Supplementary Methods).

To determine the prioritized cell types under COPD, aging, and smoking, we downsampled the data to compare the same number of cells across all cell types (see Additional file 1: Supplementary Methods) and found that monocytes had the largest number of DEGs in COPD, while club cells and macrophages were the most affected types under aging and smoking, respectively (Fig. 2E and Additional file 2: Fig. S2G). This finding was validated by AUGUR, which prioritizes cell types by quantifying the separability of perturbed and unperturbed cells within a high-dimensional space (Fig. 2F and see Additional file 1: Supplementary Methods). Note that both analyses revealed that monocytes were among the top-prioritized cell types under all conditions; therefore, they were the focus of our following analyses.

### Condition-specific status of monocytes presents snapshots of system-level malfunctions

Given the shared functions and transcriptomic similarity among cell types in the mononuclear phagocyte system (MPS), we first ruled out the possibility of clustering errors by comparing our data with 2 recently published lung scRNA-seq datasets [25, 26]. Our in-depth classification of mononuclear phagocytes was in high agreement with previous annotations (Additional file 2: Fig. S3A and see Additional file 1: Supplementary Methods), including 5 subsets of DCs (myeloid DC1s (mDC1s), myeloid DC2s (mDC2s), plasmacytoid DCs (pDCs), IGSF2 + DCs, and TREM2 + DCs), 2 major clusters of macrophages (FABP4− macrophages and FABP4+ macrophages) and particularly 3 subtypes of monocytes (CD14+ classical monocytes, CD14+ intermediate monocytes, and CD16 + non-classical monocytes) (Additional file 2: Fig. S3B-3D and Additional file 5: Dataset 3). We also noted that the non-classical monocytes were predominantly from young controls (Additional file 2: Fig. S3E); hence, there was a lack of statistical power to detect expression changes in this subtype (Additional file 2: Fig. S3F), so we restricted our analyses to two CD14+ subtypes of monocytes.

**Fig. 3:**
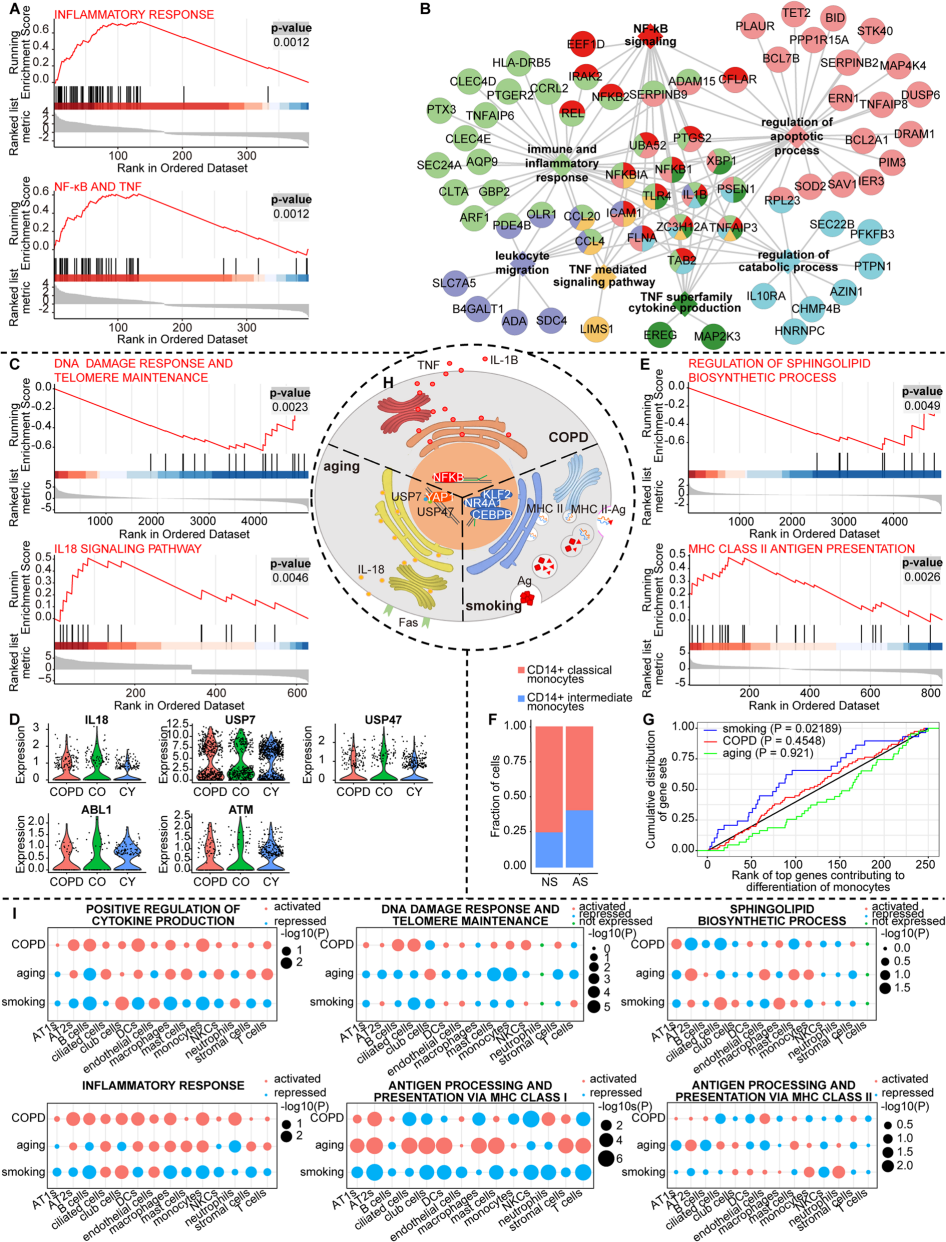
Condition-specific status of monocytes presents snapshots of system-level malfunctions. **A** GSEA analysis highlighting the inflammatory response and NF-κB as well as TNF signalings activated in monocytes under COPD. **B** Functional enrichment network generated from the up-regulated COPD-associated DEGs in monocytes. Genes (circles) are linked with the corresponding biological processes (squares in the same color) in which they are enriched. **C** GSEA analysis highlighting DNA damage response and telomere dysfunction as well as activated IL-18 signaling pathway in aged monocytes. **D** Violin plots displaying the expression distributions of *IL-18*, *USP7*, *USP47*, *ABL1*, and *ATM* in monocytes from lungs of young controls (CY), old controls (CO), and COPD patients (COPD). **E** GSEA analysis highlighting inhibited inflammatory response and activated antigen presentation via MHC class II in monocytes induced by smoking. **F** Bar plot showing the relative proportion of CD14+ classical monocytes and CD14+ intermediate monocytes across active smoker (AS) and never-smoker (NS) groups. **G** Kolmogorov–Smirnov plot showing the enrichment of COPD-, aging-, and smoking-associated DEGs of monocytes among the set of top 250 driver genes (largest waldStat from slingshot associationTest) responsible for monocytes differentiation from the CD14+ classical monocytes to the CD14+ intermediate monocytes. The one-sided (greater) Kolmogorov–Smirnov (K-S) test is used to determine the enrichment p-value. **H** Schematic diagram demonstrating specific changes of monocytes under COPD, aging, and smoking conditions. Monocytes from COPD lung tissues show aberrantly activated NF-κB signalling, along with increased expression of pro-inflammatory factors, such as IL-1β and TNF (COPD). In aged monocytes, IL-18 is significantly up-regulated due to DNA damage and telomere dysfunction, providing linkage between innate and adaptive immunity (aging). Monocytes are blocked in CD14+ intermediate sub-cluster by smoking via down-regulating differentiation driver genes, with enhanced function of antigen processing and presentation via MHC class II (smoking). **I** Dot plots showing the extent to which the biological processes of our interest are activated or repressed in all the cell types under COPD, aging, and smoking conditions by GSEA analysis. The red dot represents activation (normalized enrichment score > 0), the blue one represents repression (normalized enrichment score < 0), and the green one represents that genes involved in the biological process are not expressed in this cell type; p-value (P) is indicated by dot size

Next, we performed GSEA analysis to uncover perturbed functions associated with COPD, aging, and smoking (see Additional file 1: Supplementary Methods). Intriguingly, monocytes appeared to play very distinct immunological roles under different conditions (Additional file 2: Fig. S3G). In COPD, inflammatory responses, mediated primarily by NF-κB as well as tumor necrosis factor (TNF) signaling, were aberrantly activated in monocytes (Fig. 3A), as well as some downstream processes, such as negative regulation of apoptosis and leukocyte migration (Fig. 3B), a clear indication that monocytes function as a component of innate immunity. (Additional file 2: Fig. S3H), suggesting that at least in a subset of COPD patients, it was monocytes that triggered innate immune-driven inflammation, rather than macrophages, a long-recognized orchestrator in COPD pathogenesis. With regard to aging, inflammation appeared to be low grade, an expected phenomenon termed “inflammaging” [11], as many upstream regulators of the NF-κB and TNF pathways (such as *REL* and *MAP2K3*) were marginally increased in aged monocytes, and many pro-inflammatory effectors (such as *IL1B*, *CCL20*, and *ICAM1*) even showed decreased expression (Additional file 2: Fig. S3I and Additional file 6: Dataset 4). Notably, genes involved in interferon gamma (IFN-γ) production, including the typical IFN-γ stimulator *IL18*, were significantly up-regulated in aged monocytes (Fig. 3C, D), suggesting that interleukin-18 (IL-18)-mediated signaling is responsible for the maintenance of inflammaging. Recent studies have shown that telomere dysfunction can induce the activation of IL-18-mediated signaling via the YAP1 complex [27]. Consistently, our study showed that down-regulated DEGs in aged monocytes were enriched for DNA damage response and telomere maintenance (Fig. 3C), and components of the YAP1 complex were significantly activated, along with 2 deubiquitinases vital to both inflammasome activation and YAP1 stabilization, namely, *USP7* and *USP47* (Fig. 3D). Regretfully, the expression of *YAP1* was too low to detect (Additional file 2: Fig. S3J). Altogether, these findings provide a plausible mechanism underlying the initiation of inflammaging. Other aging-compromised functions might also contribute to inflammaging, such as translation, vesicle transport and secretion (Additional file 2: Fig. S3K), as their malfunctions could lead to retarded immune responses. Collectively, given the well-established role of IL-18 in the development of optimal T helper type 1 (Th1) cell responses via IFN-γ [28], our study revealed that monocytes link innate and adaptive immunity in aged lungs. In striking contrast to COPD and aged monocytes, monocytes in lungs from active smokers mainly functioned as antigen-presenting cells (APCs) in that genes involved in MHC class II antigen presentation were overrepresented in up-regulated DEGs when active smokers were compared with never-smokers (Fig. 3E and Additional file 2: Fig. S3L). Note that our study and others consistently defined CD14 + intermediate monocytes as APCs in the population of monocytes [29] (Additional file 2: Fig. S3C). Concordantly, we observed a large increase in the proportion of the intermediate subtype in active smokers (Fig. 3F). To rule out the possibility that the deviation in cell composition was simply a result of random sampling, we performed trajectory analysis to identify potential driver genes responsible for monocyte differentiation from the classical to the intermediate subtype (Additional file 2: Fig. S3M, S3N, Additional file 7: Dataset 5, and see Additional file 1: Supplementary Methods) and found that smoking-associated DEGs were indeed enriched among driver genes, whereas COPD- or aging-associated DEGs were not (Fig. 3G and Additional file 2: Fig. S3O). Accompanying antigen processing and presentation via MHC class II was T-cell activation, leukocyte chemotaxis, and particularly the dysregulation of the biosynthesis of sphingolipids (Additional file 2: Fig. S3G), which have gained growing attention for their increasingly recognized immunoregulatory role in inflammation, antigen presentation and thereby the pathophysiology of COPD [30–32]. We summarized the key points regarding the distinct immunological roles of monocytes in a schematic illustration in Fig. 3H. We also validated these findings by leveraging recently published COPD scRNA-seq datasets [17, 22] and showed that the combined analysis findings were in good agreement with our findings (Additional file 2: Fig. S3P, S3Q and see Additional file 1: Supplementary Methods).

With the rationale that the prioritized cell types likely responded or contributed most to the disease phenotype and therefore their status could to some extent be a proxy to uncover system-level malfunctions, we examined whether other cell types underwent similar or biologically relevant changes conditionally by using the GSEA enrichment score as a metric to measure the degree of the activation or repression of any biological process of interest. In line with our expectations, inflammatory responses and cytokine production were more prevalent in the COPD-associated environment, and antigen presentation via MHC class II was more active in smoking-associated APCs (Fig. 3I). In particular, we observed widespread activation of antigen presentation via MHC class I in aged structural cells, corresponding to the induction of Th1 cell responses mediated by aged monocytes (Fig. 3I). In summary, our analyses demonstrated that the top-prioritized cell type, herein monocytes, was indeed a crucial orchestrator in COPD pathogenesis, especially when aging and smoking were heavily involved.

### “COPD core” pathologies: altered intercellular communications and the pro-apoptotic effect of monocytes on AT2s

**Fig. 4:**
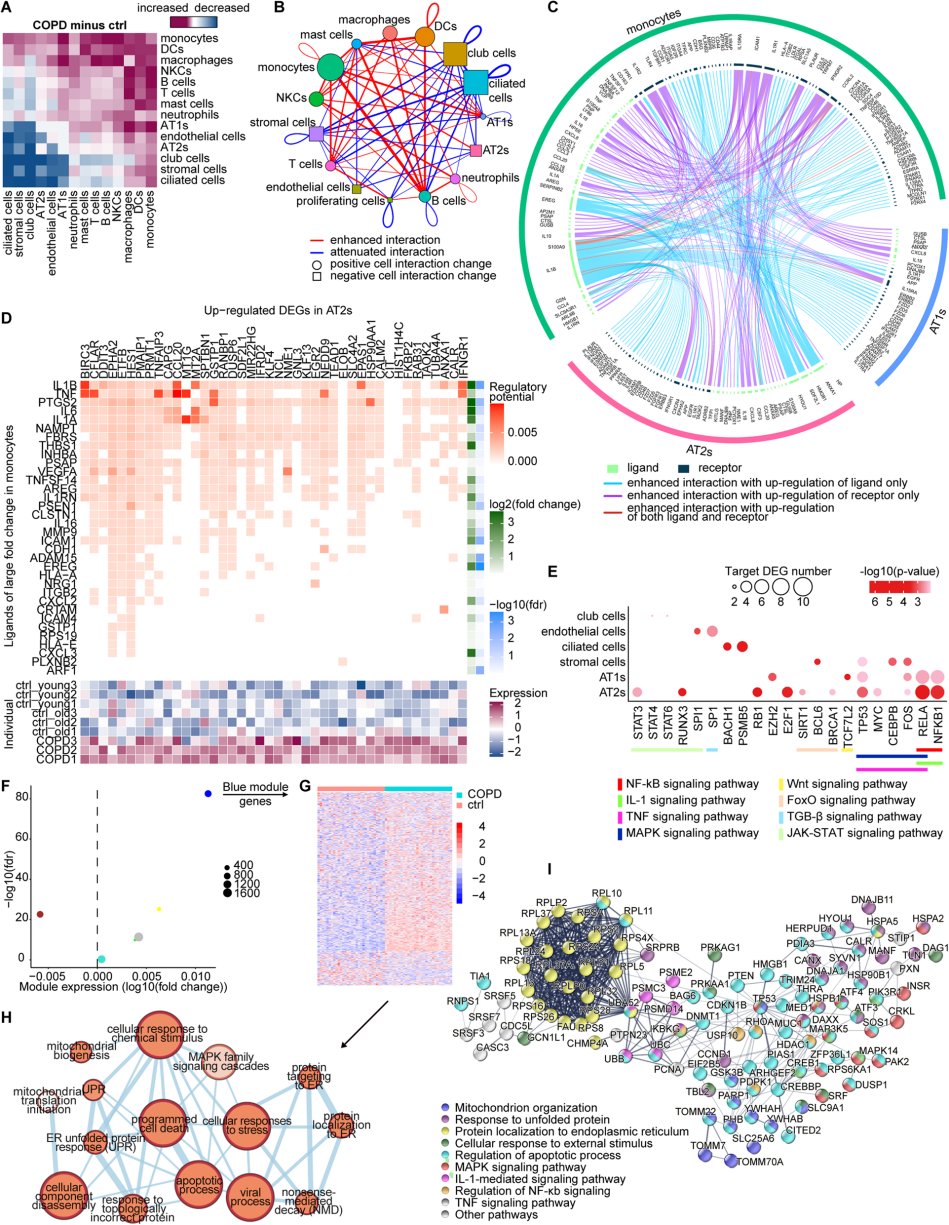
“COPD core” pathologies: Altered intercellular communications and pro-apoptotic effect of monocytes on AT2s. **A** Heatmap representing the changes of the number of ligand-receptor interactions in CCIs among 15 cell types predicted by CellPhoneDB between COPD and control lungs. The colors from red to blue represent changes of CCIs from increased to decreased. **B** Network reflecting the quantification of the changes of the ligand-receptor interactions number in CCIs among 15 cell types showed in **A**. Only the changes of interaction > 10% increase/decrease are shown in the network. Red lines connect the cells showing enhanced interactions in COPD lung, while blue lines connect those showing attenuated interactions. The thicker the line, the greater the change of interaction. Circle represents positive total changes of CCIs of a certain cell type, while square represents negative one. The larger the square or circle, the greater the total changes. Each cell type is represented by a unique color. **C** Circos plot showing the ligand-receptor pairs responsible for the enhanced CCIs among monocytes, AT1s, and AT2s predicted by CellPhoneDB, for which monocytes are either sender cells or receiver cells. Each light green inside arc represents a ligand and each dark green one represents a receptor. Blue lines link ligands and receptors showing enhanced interactions with up-regulation of ligands only. Purple lines link ligands and receptors showing enhanced interactions with up-regulation of receptors only. Red lines link ligands and receptors showing enhanced interactions with up-regulation of both ligands and receptors. **D** The top heatmap showing the potential of ligands of large fold change (log2(fold change) > 0.14 or log2(fold change) < − 0.14) in monocytes (on the y-axis) regulating the up-regulated COPD-associated DEGs in AT2s (on the x-axis) predicted by NicheNet analysis. The fold change and false discovery rate of these ligands are shown in 2 separate heatmaps on the right. The mean gene expressions of the up-regulated COPD-associated DEGs in AT2s across 9 samples are shown in the lower heatmap. The mean gene expression per sample is represented as a z transformed value (across all samples). **E** Dot plot showing the number of predicted target DEGs of the potential upstream transcription factors on the x-axis in the given cell types on the y-axis. The potential upstream transcription factors are grouped by their pathway annotations which are represented by lines in different colors. p-values are indicated by dot color; The numbers of target DEGs are indicated by dot size. **F** Scatterplot showing the co-expression gene modules of AT2s detected by WGCNA analysis. The x axis represents the log2(fold change) of modules’ expression under COPD, and y axis shows the statistical significance of modules’ association with COPD. Each circle represents a module, whose size is proportional to the number of genes in the module. **G** Heatmap showing relative expression of 838 genes in Blue module. All cells within COPD or control are randomly clustered into 30 groups, and then averages of expression are calculated within each group, resulting in 30 transcriptomic profiles each for COPD and control, which displays a distinct overall up-regulation in COPD lungs. **H** Enrichment network demonstrating functional topologies among major biological processes of Blue module. Each biological process is represented by a node whose size is proportional to the number of genes enriched in the biological process and color darkness is proportional to enrichment significance. The connecting line represents the overlapping genes between two biological processes, whose thickness is proportional to the percentage of overlapping. **I** Functional interaction network is constructed for Blue module genes enriched in more than 2 biological processes in **H** by STRINGDB with active interaction source from experiments only. Each node represents a protein with its encoding gene labelled. Each edge represents the interaction between the two proteins. Line thickness indicates the strength of data support. Proteins are clustered according to their pathway annotations which are reflected by the colors of circles. Disconnected genes are not shown

The activation of pro-inflammatory signaling pathways and cytokine production in COPD-associated monocytes suggested that this prioritized cell type likely drives transcriptional alterations in other cell types via intercellular communication. We therefore performed connectome analyses to identify differences in CCIs between control and COPD lungs. More specifically, we quantified changes in CCIs by measuring differences in the numbers of CellPhoneDB-predicted ligand-receptor pairs among the 15 cell types (see Additional file 1: Supplementary Methods). Overall, CCIs between innate immune cells and non-immune structural cells were increased, which was particularly obvious for monocytes (Fig. 4A and Additional file 2: Fig. S4A), strongly supporting their vital role in modulating microenvironmental signals in COPD lungs. We then focused on CCIs with a > 10% increase in predicted ligand-receptor interactions from control to COPD lungs (Fig. 4B). Interestingly, both types of alveolar epithelial cells (AT1s and AT2s) exhibited the largest enhanced interactions with monocytes (Fig. 4B). A Circos illustration of ligand-receptor pairs responsible for elevated CCIs between monocytes and alveolar epithelial cells as a result of significant expression changes highlighted several pro-inflammatory cytokines up-regulated in COPD-associated monocytes, including IL-1β, TNF, and EREG (Fig. 4C and see Additional file 1: Supplementary Methods). However, the Circos illustration of ligand-receptor pairs accounting for enhanced CCIs between monocytes and other immune cells revealed that the most extensively involved ligands and receptors were all from COPD-associated monocytes, including chemokines (*CCL20* and *CCL4*), adhesion molecules (*ICAM1*), and chemokine receptors (*CCRL2* and *CXCR4*) (Additional file 2: Fig. S4B). These findings implied that in addition to being reactive to surrounding cells, monocytes within COPD lung tissues dominated their enhanced interactions with both structural cells and other immune cells. Such frequent intercellular communications relying on pro-inflammatory ligand-receptor pairs fit well with the role of monocytes in orchestrating the inflammation core to COPD pathogenesis. To further explore the downstream effects of the interactions between monocytes and alveolar epithelial cells, we performed NicheNet analysis to prioritize ligands that could best predict the DEGs in COPD-associated alveolar epithelial cells (see Additional file 1: Supplementary Methods). Among the top-prioritized ligands, IL-1β and TNF were predicted to be influential upstream ligands that could regulate many genes involved in the NF-κB and mitogen-activated protein kinases (MAPK) pathways in both alveolar epithelial cell types (Fig. 4D and Additional file 2: Fig. S4C). In accordance with this finding, the upstream analysis by Metascape predicted that several transcription factors in NF-κB, IL-1, TNF, and MAPK signaling pathways could potentially have regulated the identified DEGs in AT1s and AT2s (Fig. 4E and see Additional file 1: Supplementary Methods). Collectively, our analyses suggested that elevated CCIs due to activation of pro-inflammatory signaling pathways in COPD-associated monocytes, especially IL-1 and TNF signaling, might be major drivers of alveolar epithelial inflammation in COPD.

Next, to verify the predicted pro-inflammatory effect of monocytes and further clarify the biological consequences of such interactions on alveolar epithelial cells, we performed an in-depth analysis of the transcriptomic changes in alveolar epithelial cells, with a specific focus on AT2s, which ranked second only to monocytes in their contribution to COPD heritability (Fig. 2D) and showed more than 10% enhanced interaction with monocytes uniquely among all cell types in COPD lung tissues (Fig. 4B). To better characterize signaling topologies, we performed WGCNA analysis to find COPD-associated gene clusters with highly correlated expression levels and found a module, referred to as the Blue module, of 838 genes whose overall expression was significantly up-regulated in COPD (Fig. 4F, G, Additional file 8: Dataset 6, and see Additional file 1: Supplementary Methods). We used an enrichment map to reveal functional topologies among major biological processes of the Blue module (Fig. 4H), including external stimuli (cellular response to chemical stimulus), cellular damage (apoptotic process and programmed cell death), and signaling cascades in accordance with those predicted in Fig. 4E (MAPK family signaling cascades), strongly attributing the cellular damage in AT2s to the external immune stimulus from monocytes mediated by pro-inflammatory ligands such as IL-1β and TNF. Subsequently, we constructed a functional interaction network by STRINGDB for the proteins encoded by Blue module genes that were enriched in more than 2 biological processes in Fig. 4H to better demonstrate the key biological processes in COPD-associated AT2s as well as their interrelationships (Fig. 4I and see Additional file 1: Supplementary Methods). Notably, the STRINGDB network not only emphasized the biological processes (such as cellular response to external stimulus, response to unfolded protein, mitochondrion organization, and apoptotic processes) most associated with COPD in AT2s but also contained the predicted signaling pathways (including NF-κB, IL-1, TNF, and MAPK signaling pathways) in AT2s influenced by ligands from monocytes (Fig. 4E). More importantly, this network clearly showed that external stimuli from monocytes, especially represented by IL-1β and TNF, led to the apoptosis of AT2s via endoplasmic reticulum (ER) stress, mitochondrial dysfunction, and the MAPK signaling pathway, wherein several hub proteins were linked by NF-κB signaling (Fig. 4I).

When exploring the possibility that cellular damage in AT2s might compromise their capacities to differentiate into AT1s [33] by slingshot (Additional file 2: Fig. S4D), inspired by the observation that AT2s from COPD lung tissues were more clustered in the early stage of the differentiation path (Additional file 2: Fig. S4E), we further identified potential driver genes responsible for AT2 differentiation and indeed observed an overrepresentation of COPD-associated DEGs in monocytes among them, as well as aging-associated DEGs (Additional file 2: Fig. S4F and see Additional file 1: Supplementary Methods). Notably, a number of pro-inflammatory and pro-apoptotic genes were identified as driver genes, along with a recently discovered AT2 surface marker, *CD74*, which is helpful for discriminating AT2s from AT1s [33] (Additional file 2: Fig. S4G and Additional file 9: Dataset 7), implying that differentiation suppression of COPD-associated AT2 might be partly accounted for by inflammation and apoptosis.

In summary, our analyses established a signaling pathway underlying the “COPD core” pathogenesis, which started from pro-inflammatory factors secreted by monocytes, transmitted signals by CCIs, and ultimately resulted in the apoptosis of AT2s as well as restrained differentiation towards AT1s and hence the potential failure of alveolar epithelial repair after injury.

### Potential mechanism of aging facilitating COPD development: an autoimmune airway niche modulated by aged club cells underlying the injury of airway epithelia

**Fig. 5:**
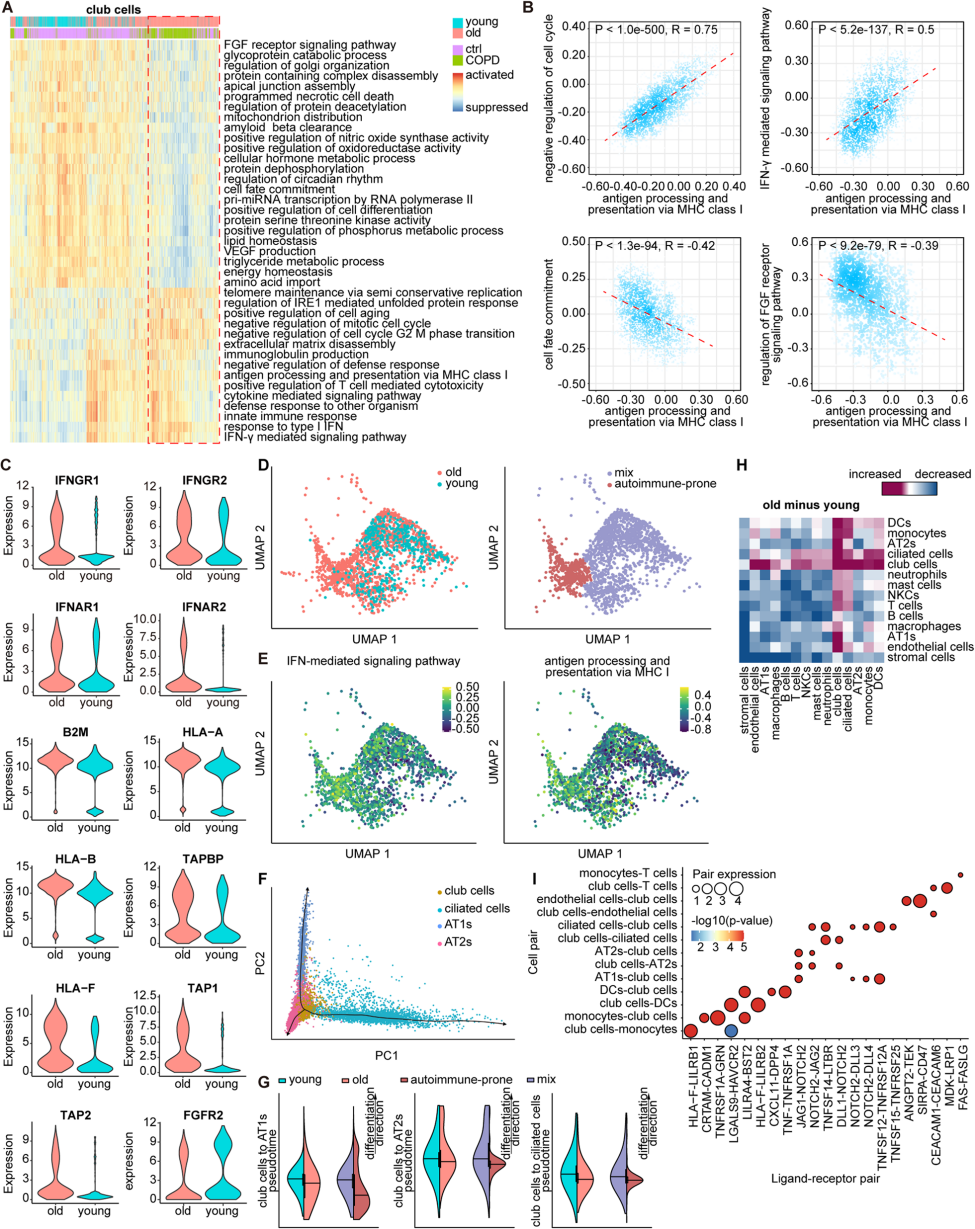
Potential mechanism of aging facilitating COPD development: An autoimmune airway niche modulated by aged club cells underlying the injury of airway epithelia. **A** Heatmap showing GSVA analysis enrichment scores of the top altered biological pathways in club cells from lung tissues of young (cyan) and old (pink) individuals. The colors from red to blue represent alterations of biological pathways states from activated to suppressed. The disease conditions of individuals from which club cells were derived are reflected as different colors of the bar above the heatmap. A distinct subset of club cells characterized by hallmarks of aging are framed by a red dotted rectangular box in the heatmap, which are mainly derived from COPD lungs. **B** Scatterplots showing the correlations of GSVA analysis enrichment score between pathway of antigen processing and presentation via MHC class I and four other biological pathways (including negative regulation of cell cycle, IFN-γ-mediated signaling pathway, cell fate commitment, and regulation of FGF receptor signaling pathway) in club cells. P: Pearson correlation p-value of two-sided paired t-tests. R: Pearson correlation coefficient of two-sided paired t-tests. **C** Violin plots for genes encoding IFN receptors (*IFNRG1*, *IFNRG2*, *IFNRA1*, and *IFNRA2*), genes involved in pathway of antigen processing and presentation via MHC class I (*B2M*, *HLA-A*, *HLA-B*, *HLA-F*, *TAP1*, *TAP2*, and *TAPBP*), and *FGFR2* (FGF receptor 2) in club cells from lung tissues of young (cyan) and old (pink) individuals. **D** UMAP plot showing all the club cells from lung tissues of young (cyan) and old (pink) individuals (left). All the club cells are classified in depth into several sub-clusters, among which one sub-cluster highly aggregated in the club cells from lung tissues of old individuals is named as autoimmune-prone sub-cluster (brown). Others are together referred to as mix sub-cluster (purple). UMAP plot showing the two sub-clusters of all the club cells (right). One dot represents a club cell. **E** Feature plots for GSVA analysis enrichment scores of IFN-γ-mediated signaling pathway (left) and antigen processing and presentation via MHC class I (right) in all the club cells. One dot represents a club cell. The colors from yellow to dark green represent alterations of biological pathways states from activated to suppressed. **F** A predicted trajectory of airway epithelial cells differentiation including club cells (yellow), ciliated cells (green), AT1s (purple), and AT2s (red). Each point corresponds to a single cell. **G** Violin plot of club cells from lung tissues of young and old individuals as well as autoimmune-prone sub-cluster of club cells and mix sub-cluster of club cells on differentiation of club cells to AT1s (left), club cells to AT2s (middle), and club cells to ciliated cells (right) pseudotime. **H** Heatmap representing the changes of CCIs among 15 cell types predicted by CellPhoneDB between young and old lungs. The colors from red to blue represent changes of CCIs from increased to decreased. **I** Overview of selected ligand-receptor pairs (x-axis) predicted to be responsible for the enhanced interactions between the given cell pairs (y-axis) in aged lungs, compared to young lungs. Each circle represents an interacting ligand-receptor pair. The p-values of CellPhoneDB CCIs are indicated by colors of circles. The means of the average expression levels of interacting ligand in sender cells and interacting receptor in receiver cells are indicated by circle size

Since club cells were the most prioritized aging-associated cell type (Fig. 2E and F), we focused our attention on club cells to illustrate the potential mechanism by which aging might facilitate COPD development. Prevalent overexpression of MHC class I molecules hinted at increasing susceptibility to autoimmunity during aging (Fig. 3I). Hence, to better characterize the cellular status of each single cell, we performed GSVA analysis to assess single-cell functional enrichment (see Additional file 1: Supplementary Methods). The heatmap listing the top altered biological terms between club cells from lungs of the elderly and young individuals revealed a distinct subset of aged club cells characterized by hallmarks of aging, such as a loss of proteostasis (regulation of IRE1 mediated unfolded protein response) and cellular senescence (negative regulation of mitotic cell cycle), which were mainly derived from COPD lungs (Fig. 5A). Similarly, other airway epithelial cells, including AT1s, AT2s, and ciliated cells, showed signs of these hallmarks with age (Additional file 2: Fig. S5A–S5C). Note that single-cell functional enrichment of the MHC class I antigen presentation pathway was highly correlated with those of negative regulation of the cell cycle and IFN-γ-mediated signaling pathway among all 4 types of airway epithelial cells (Fig. 5B and Fig. S5D–S5F), strongly suggesting a potential association between autoimmune susceptibility as well as IFN reactivity and cellular senescence of epithelial cells and COPD pathology.

Genes encoding IFN receptors (including *IFNGR1*, *IFNGR2*, *IFNAR1*, and *IFNAR2*) were significantly increased in airway epithelial cells with age (Fig. 5C and Additional file 2: Fig. S5G–S5I), and the up-regulation of these IFN receptors together with their ligands was also observed in the aged nervous system [34–36], which reportedly resulted in an increased level of MHC class I molecules in aged neurons [34]. Correspondingly, vital components of the MHC I system, especially β2-microglobulin (*B2M*), showed a general up-regulation in aged airway epithelial cells (Fig. 5C and Additional file 2: Fig. S5J-5L), which could injure lung epithelia by MHC class I-mediated cytotoxicity and directly induce epithelial cell senescence, as previously reported [37]. Particularly, in club cells, we also observed negative correlations between MHC class I antigen presentation and cell fate commitment as well as the fibroblast growth factor (FGF) receptor signaling pathway, which is indispensable to club cell proliferation and differentiation [38] (Fig. 5B). The expression of *FGFR2* indeed declined in aged club cells (Fig. 5C). Given that club cells are multifunctional bronchoalveolar epithelial progenitor cells that are of great importance to airway epithelium maintenance [39], such negative correlations raised the possibility that the autoimmunity of aged club cells may compromise their stemness through the modulation of FGF signaling, as was reported in other types of progenitor cells and stem cells [35]. Concordantly, we further identified an autoimmune-prone sub-cluster unique to aged club cells (Fig. 5D, Additional file 2: Fig. S5M, S5N, and Additional file 10: Dataset 8) and enriched in COPD lungs (Fig. 5A), for which antigen presentation via MHC I and IFN-γ-mediated signaling pathway were remarkably activated (Fig. 5E), whereas cell proliferation and the FGF receptor signaling pathway were suppressed (Additional file 2: Fig. S5O and S5P) compared to other aged club cells. Next, after dissecting the inferred differentiation trajectories from club cells to other epithelial cells (Fig. 5F, Additional file 2: Fig. S5Q, Additional file 11: Dataset 9, and see Additional file 1: Supplementary Methods), we found that aged club cells were more clustered in the earlier phase in all three differentiation modes and even more so for the autoimmune-prone sub-cluster of aged club cells (Fig. 5G). In addition, we observed an overrepresentation of genes regulating proliferation among differentiation driver genes, as well as aging-associated DEGs related to proliferation, such as down-regulated pro-proliferative DEGs (including *SOX2, TMEM45A, CRNDE,* and *CD55*) and up-regulated anti-proliferative DEGs (including *IGFBP7* and *TIMP2*) in aged club cells, the degree of which was even greater in the autoimmune-prone sub-cluster (Additional file 2: Fig. S5R). Collectively, we revealed an autoimmunity-stimulating IFN/MHC I axis generally activated in aged lung epithelial cells, which both directly led to epithelial cell injury and specifically dampened the stemness of club cells by inhibiting FGF signaling.

Since our data demonstrated cellular senescence and an autoimmune response in non-immune structural cells from aged lungs, we further investigated whether and how other cell types were involved in these processes by analysing the CCIs. Intriguingly, aged club cells displayed the strongest enhanced communications with other cell types (Fig. 5H), suggesting a non-negligible role of this aging-associated prioritized cell type in modulating the microenvironment that increases susceptibility to COPD. Hence, we took a deeper look into the ligand-receptor pairs responsible for enhanced CCIs between club cells and other cell types (Fig. 5I). In general, cytotoxic responses to senescent cells were augmented in aged immune cells, particularly monocytes, DCs, NKCs and T cells, supported by significantly increased expression of ligands that promote CD8^+^ T-cell and NKC cytotoxic responses, such as *CRTAM*, *HAVCR2*, *TNFRSF1A*, *BST2*, and *FAS*. It is noteworthy that aged monocytes displayed the largest increase in CCIs with aged club cells (Fig. 5H), suggesting that the possible monocyte recruitment of aged club cells may further amplify the epithelial damage attributed to autoimmune responses and that monocytes may simultaneously elevate the autoimmunity of aged club cells in other direct ways in addition to IL-18. Aged club cells also presented enhanced NOTCH2-mediated interactions with other epithelial cells (Fig. 5I), implying that other types of aged epithelial cells might prevent the replenishment of themselves from club cells by changing the differentiation direction of club cells into goblet cells [40], a cell type not detected in our data. With regard to CCIs between club cells and endothelial cells, we proposed that the increased secretion of CD47 [41] and carcinoembryonic antigen-related cell adhesion molecule 1 (CEACAM1) [42] from aged club cells was likely to promote vascular damage and aging processes. In summary, these findings illustrated an autoimmune airway niche during aging responsible for the cytotoxic damage of airway epithelia, which was triggered by the autoimmunity-inducing IFN/MHC I axis, further augmented by monocytes, and modulated by aged club cells.

### Potential mechanism of smoking facilitating COPD development: alveolar impairment due to detrimental intercellular communication between macrophages and endothelial cells

**Fig. 6:**
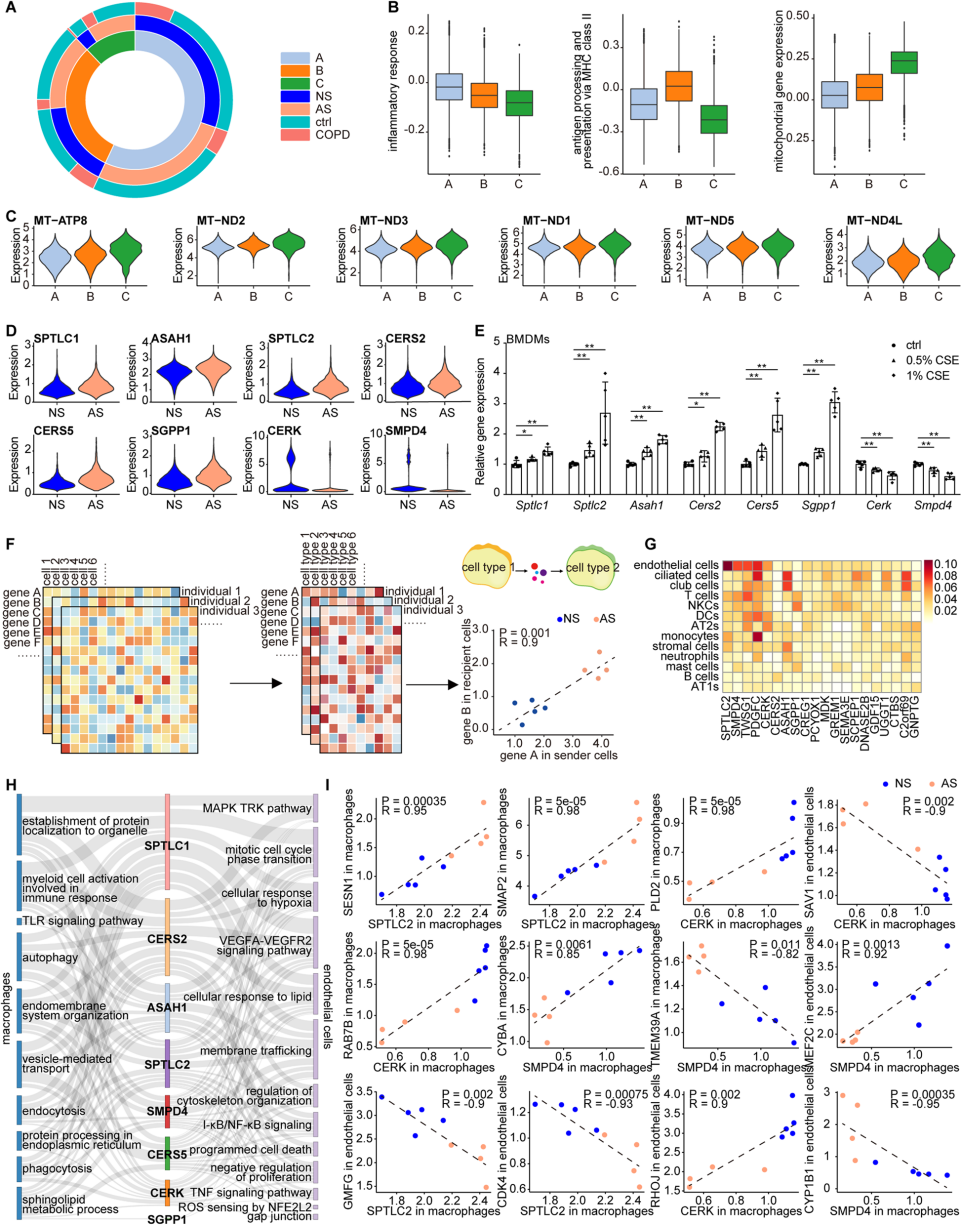
Potential mechanism of smoking facilitating COPD development: Alveolar impairment due to the detrimental intercellular communications between macrophages and endothelial cells. **A** The sunburst diagrams illustrating the cell number proportion of active smoker (AS)/never-smoker (NS) and COPD/control in the macrophage three subtypes (A, B, and C). The inner ring represents the cell number proportion of macrophage subtype A, B and C in macrophage. The middle ring represents the cell number proportion of active smoker and never-smoker in macrophage subtype A, B and C. The outer ring represents the cell number proportion of COPD and control in macrophage subtype A from active smoker (AS)/never-smoker (NS), macrophage subtype B from active smoker (AS)/never-smoker (NS) and macrophage subtype C from active active smoker (AS)/never-smoker (NS). **B** Box plots showing the GSVA analysis enrichment score of inflammatory response, antigen processing and presentation via MHC class II, and mitochondrial gene expression in A, B, and C subtypes of macrophages. **C** Violin plots for the expression of genes involved in mitochondrial electron transport including *MT-ATP8*, *MT-ND2*, *MT-ND3*, *MT-ND1*, *MT-ND5*, and *MT-ND4L* in subtypes A, B, and C of macrophages. **D** Violin plots for the expression of 8 genes encoding enzymes implicated in sphingolipid metabolism (*SPTLC1*, *SPTLC2*, *CERS2*, *CERS5*, *ASAH1*, *SGPP1*, *CERK*, and *SMPD4*) in macrophages from active smokers (AS) and never-smokers (NS). **E** Bone marrow-derived macrophages (BMDMs) from C57BL/6 mice were treated with (0.5% CSE, gray bars; 1% CSE, black bars) or without (ctrl, white bars) cigarette smoke extract (CSE) for 24 h, when the mRNA levels of *SPTLC1*, *SPTLC2*, *CERS2*, *CERS5*, *ASAH1*, *SGPP1*, *CERK*, and *SMPD4* were measured by qPCR in the BMDMs for each group (*P < 0.05 vs. ctrl, **P < 0.01 vs. ctrl). **F** Schematics of the correlation analysis we designed based on summarized expression profiles at the individual level between macrophages and other cell types. Cell-type-specific mean gene expression within each individual is first calculated. As a result, each individual has an expression matrix with rows corresponding to genes and columns corresponding to cell types. Next, Spearman correlation analysis between mean expression values of gene A of all individuals in sender cell type 1 and mean expression values of gene B of all individuals in recipient cell type 2 is performed to examine whether gene B in recipient cells changes coordinately with gene A dysregulated in sender cells. Finally, the correlation between the mean expression values at individual level of gene A in sender cells and the mean expression value at individual level of gene B in recipient cells across active smokers (AS) and never-smokers (NS) is visualized as scatterplot. **G** Heatmap showing the proportion of the number of genes with a correlation no less than 0.8 (Spearman rank correlation coefficient ≥ 0.8 or ≤ − 0.8) with each macrophage significantly up-regulated ligand gene expression and dysregulated sphingolipid metabolic enzyme gene expression to the number of expressed genes in each cell type. Each row is a cell type, and each column is a differential expressed ligand/enzyme gene in macrophages. White indicates low proportion and red indicates high proportion. Rows are ordered from top to bottom by the mean proportion of genes highly correlated with differential expressed ligands and enzymes in macrophages in each cell type. **H** Sankey plot demonstrating the enriched biological processes among the correlated genes in macrophages (left) and endothelial cells (right) to the smoking-associated dysregulated enzymes that participate in sphingolipid metabolism in macrophages (centre). **I** Scatter plots showing the cell-type-specific mean gene expression within each individual correlations between certain sphingolipid metabolic enzyme genes in macrophages and several representative highly correlated genes in macrophages or endothelial cells across active smokers (AS) and never-smokers (NS)

GSEA highlighted the activation of antigen presentation via MHC class II among innate immune cells (mainly monocytes, macrophages, DCs, and neutrophils) in the lungs from active smokers (hereafter referred to as “smoking lungs”) (Fig. 3I). A deeper examination of the functional enrichment of these cells under smoking by GSVA confirmed this observation, as many processes indispensable to MHC class II trafficking and processing were also enriched, such as endocytosis and vesicle transportation (Additional file 2: Fig. S6A–S6D). We also noted that macrophages, the top-prioritized cell type associated with smoking, appeared to be much more heterogenous than other cell types (Additional file 2: Fig. S6A), partly because this cell type was most abundantly sampled in our dataset. Hence, differential expression-based analysis was not adequate to explore alterations in macrophages. We therefore addressed heterogeneity by k-means clustering based on the top 400 highly variable GSVA terms and segregated macrophages into 3 subtypes (A, B, and C) (Fig. 6A and see Additional file 1: Supplementary Methods). The GSVA enrichment of subtypes A and B fit well with a role in the innate immune response and antigen presentation via MHC class II, respectively, thereby largely corresponding to the homeostatic function of macrophages (Fig. 6B). Subtype C, however, was distinguished by the expression of genes involved in mitochondrial electron transport (Fig. 6C), suggesting a distinctive cellular metabolism of this subtype. Intriguingly, subtype C comprised a disproportionate number of cells from active smokers (odds ratios (ORs) > 3.5, p-value < 1.1e−172, one-sided Fisher’s exact test, alternative = “greater”; Fig. 6A). Moreover, subtype C was nearly absent in never-smoking COPD patients (ORs < 0.51, p-value < 7.8e−09, one-sided Fisher’s exact test, alternative = “less”; Fig. 6A), strongly suggesting that subtype C might be an important smoking-induced subtype of macrophages that contributes to COPD pathogenesis.

Along with mitochondrial gene expression, we also observed an overexpression of several genes encoding enzymes implicated in sphingolipid metabolism in subtype C macrophages (Additional file 2: Fig. S6E). Inspired by the pleiotropic effect of bioactive sphingolipids on multiple cell types in the lung [43], we sought to determine whether and how sphingolipid metabolism in macrophages plays a role in smoking lungs. In addition to macrophages, altered sphingolipid metabolism may affect endothelial cells, which was implicated by the enrichment of smoking-associated DEGs from endothelial cells in “cellular response to lipid” (Fig. 2B). Indeed, we found evidence for enhanced pro-inflammatory sphingolipid biosynthesis (including ceramide and sphingosine) along with the diminished biosynthesis of anti-inflammatory sphingolipids (including sphingosine-1-phosphate and ceramide-1-phosphate) in macrophages from smoking lungs, as demonstrated by increased macrophage expression of *SPTLC1*, *SPTLC2*, *CERS2*, *CERS5*, *ASAH1*, and *SGPP1* as well as decreased expression of *CERK* and *SMPD4* in smoking lungs (Fig. 6D). Additionally, such expression changes in these genes were observed in cigarette smoke extract (CSE)-treated bone-marrow-derived macrophages (BMDMs) from C57BL/6 mice (Fig. 6E and see Additional file 1: Supplementary Methods), further supporting pro-inflammatory sphingolipid biosynthesis in macrophages from smoking lungs.

Next, given the vesicle-transportation-dependent paracrine activities of ceramide and sphingosine reported in other tissues [44] and their detrimental role in lungs exposed to cigarette smoke [43], we hypothesized that excessive ceramide and sphingosine biosynthesized by macrophages could also have an effect on endothelial cells in smoking lungs. However, such intercellular communications based upon sphingolipid metabolites could not be assessed by the analytic strategy relying on known ligand-receptor pairs. Hence, we designed a novel approach to infer the potential contribution of activated sphingolipid biosynthesis in smoking macrophages to transcriptomic alterations in other cell types, which takes ligand-receptor pair-mediated CCIs into account at the same time due to the participation of the other 2 subtypes of macrophages in innate and adaptive immune responses (Fig. 6B). Briefly, we first summarized cell-type-specific gene expression at the individual level, and then we examined whether the expression of significantly dysregulated sphingolipid metabolic enzymes as well as up-regulated ligands in smoking macrophages changed concordantly with smoking-associated DEGs in other cell types across individuals (Fig. 6F and see Additional file 1: Supplementary Methods). We found the highest concordance of expression change between smoking-associated DEGs in endothelial cells and the abovementioned dysregulated sphingolipid metabolic enzymes and ligands in macrophages (Fig. 6G). Note that the sphingolipid biosynthesis process was not activated in endothelial cells, which ruled out the autocrine effect of sphingolipids secreted by endothelial cells (Additional file 2: Fig. S6F–S6H). To gain insight into the mechanism underpinning such metabolic communication, we sought to identify correlated genes across individuals with the 8 dysregulated sphingolipid metabolic enzymes in macrophages not only in endothelial cells to infer cell-nonautonomous effects but also in macrophages to reflect cooperative cell-autonomous alterations (Additional file 12: Dataset 10). As seen from the Sankey plot in Fig. 6H, the enriched biological processes among the correlated genes to these enzymes in macrophages included autophagy, vesicle transport, and endomembrane system organization, while those in endothelial cells included membrane trafficking, cellular response to lipid, and cytoskeleton organization, indicating that pro-inflammatory sphingolipid-based metabolic communication is likely to be transmitted in the form of vesicles from macrophages to endothelial cells and may have a damaging effect on both cell types in smoking lungs. Several enzymes and their highly correlated genes from both macrophages and endothelial cells are shown in Fig. 6I. On the other hand, we applied the same strategy to prioritize up-regulated ligands in smoking macrophages that could best account for expression changes in endothelial cells (see Additional file 1: Supplementary Methods). Specifically, we first compiled lists of highly correlated genes in endothelial cells with each smoking-associated up-regulated ligand in macrophages across individuals, and then we examined the functional overlap of each list with the smoking-associated DEGs of endothelial cells (Additional file 2: Fig. S6I). Two ligands in macrophages, namely, *PDGFC* and *TWSG1*, showed remarkable functional overlap with the smoking-associated DEGs of endothelial cells (Additional file 2: Fig. S6I), underscoring the importance of these two ligands in mediating the effect of macrophages on endothelial cells under smoking. Similarly, *PDGFC*, *TWSG1* and their representative correlated genes from endothelial cells are shown in Additional file 2: Fig. S6J.

Overall, these findings represented a scenario in which alveolar impairment by smoking was mediated by APCs due to their enhanced capacity for antigen presentation and heightened intercellular communication between macrophages and endothelial cells in both ligand- and sphingolipid-metabolite-dependent manners, which may exacerbate COPD.

### Validation of findings under the three pathological conditions by a meta-analysis

**Fig. 7:**
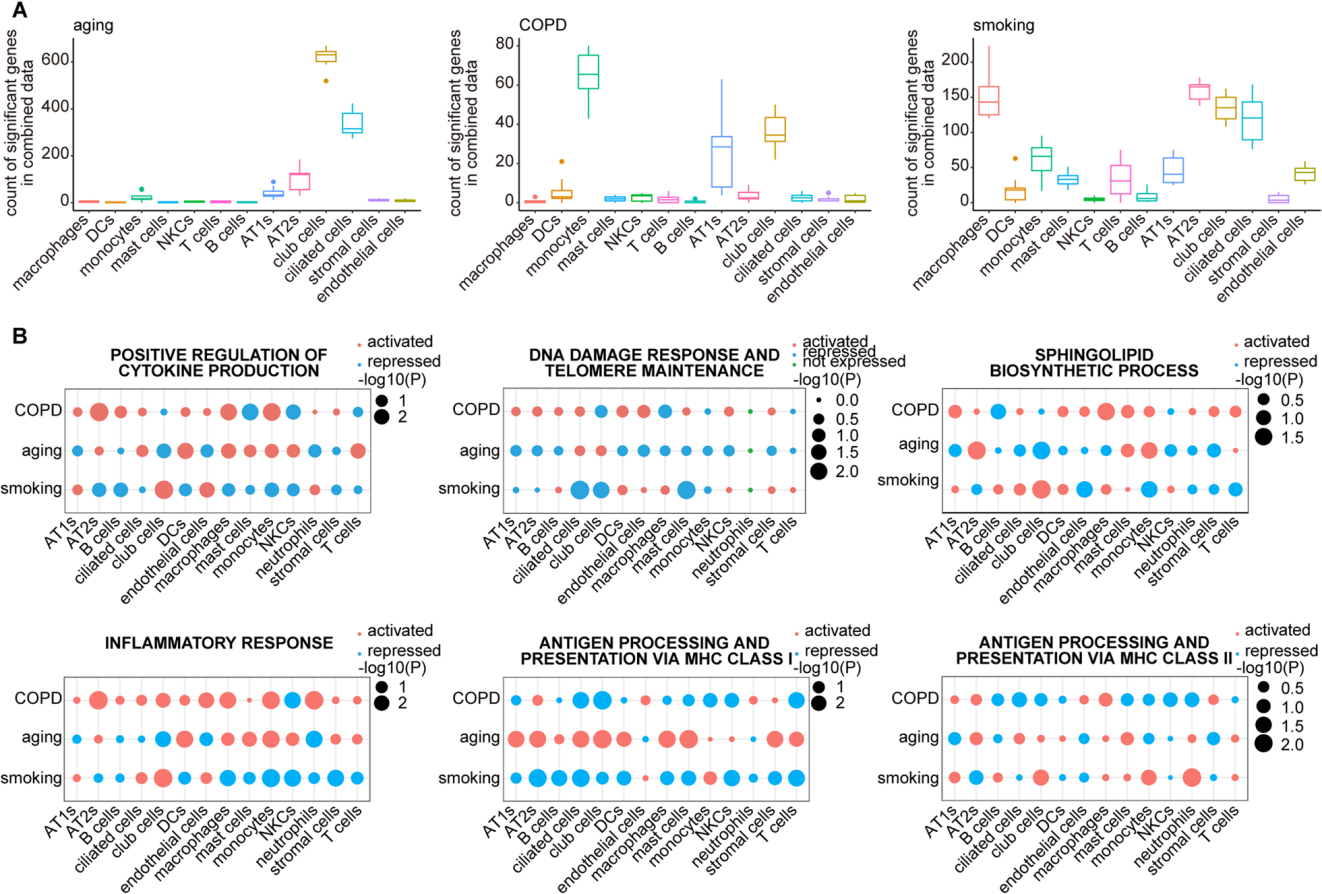
Validation of findings under three pathological conditions by meta-analysis. **A** Box plots showing the number of aging- (left), COPD- (middle), and smoking-associated DEGs (right) for all cell types except neutrophils (Adams’ scRNA-seq data do not contain neutrophils) normalized by the number of cells in downsampling analysis of the 9 + 9 dataset. **B** Dot plots showing the extent to which the biological processes of our interest in Fig. 3I are activated or repressed in all the cell types under COPD, aging, and smoking conditions by GSEA analysis of the 9 + 9 dataset. The red dot represents activation (normalized enrichment score > 0), the blue one represents repression (normalized enrichment score < 0), and the green one represents that genes involved in the biological process were not expressed in this cell type; p-value (P) is indicated by dot size

Given the high clinical heterogeneity of COPD and the single-centre source of the lung tissue samples in our study, we sought to confirm our findings, especially those of system-level malfunctions centred on prioritized cell types under three conditions (COPD, aging, and smoking), in publicly available COPD datasets (see Additional file 1: Supplementary Methods). To this end, we first combined the scRNA-seq dataset of 9 samples from both healthy and COPD individuals with different ages, races, and smoking histories (Additional file 13: Dataset 11) recently published by Adams et al*.* [22] with our scRNA-seq dataset, forming a mixed dataset (hereafter referred to as the “9 + 9 dataset”). We then repeated the downsampling analysis on the 9 + 9 dataset. Once again, monocytes and club cells were prioritized in this combined dataset under COPD and aging conditions, respectively (Fig. 7A). However, the result under smoking conditions in the 9 + 9 dataset differed slightly from that in our dataset (Fig. 7A), which was largely due to the heterogeneity of macrophages in both datasets. This result demonstrates the rationality and necessity of our analysis strategy being based on the GSVA enrichment score for subtype classification of macrophages, which can be extended to investigate the influence of smoking on macrophages in the 9 + 9 dataset in the near future. Similarly, we also measured the activation or repression states of the same biological processes in Fig. 3I across all the cell types under the three conditions in the 9 + 9 dataset by using GSEA enrichment scores (Fig. 7B). Although the 9 + 9 dataset did not fully recapitulate the status of every biological process in our dataset, these datasets showed substantial overlap in heightened inflammatory responses together with the cytokine production prevalent in monocytes and AT2s in COPD lung tissues and shared the general activation of antigen presentation via MHC class I along with DNA and telomere dysregulation among structural cells in aged lung tissues. The states of biological processes were more heterogeneous under the condition of smoking than under aging and COPD due to the high complexity in the impact of smoking, although sphingolipid biogenesis in smoking-associated macrophages as well as MHC class II-mediated antigen presentation among smoking-associated APCs were still highlighted in the 9 + 9 dataset.

Overall, this integrated analysis of published scRNA-seq data indicated that the conditional prioritized cell types and system-level malfunctions reflected by the dysregulated biological processes we found were present in people with different genetic backgrounds and clinical contexts, confirming the reliability and reproducibility of our results.

## Discussion

**Fig. 8:**
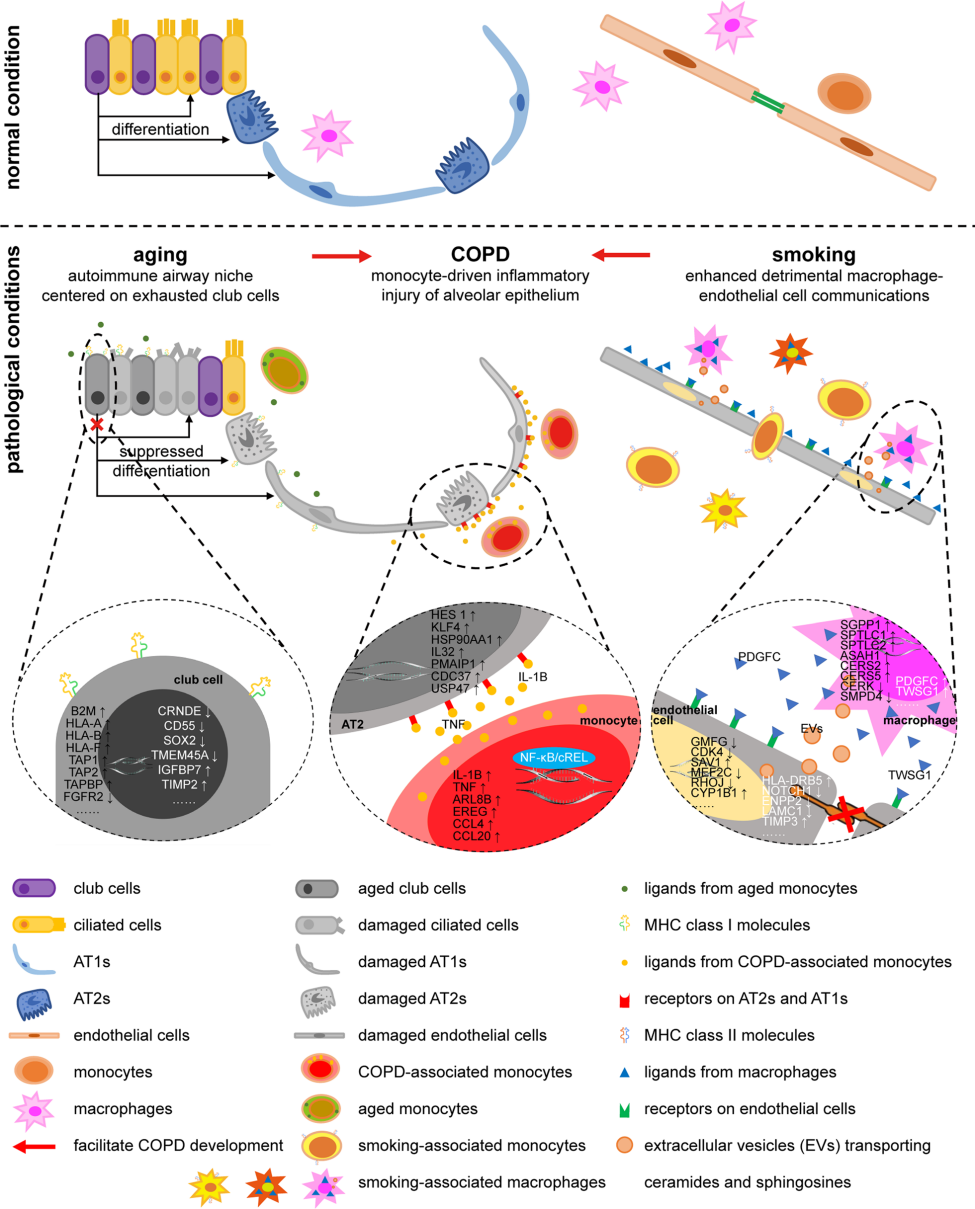
Schematics of the clarified COPD pathological model derived from our human COPD lung single-cell atlas. Monocytes orchestrate the “COPD core” pathogenesis, which starts from pro-inflammatory factors synthesized and secreted by monocytes, transmits via CCIs, and eventually leads to inflammatory injury of alveolar epithelia. In aged lungs, the IFN/MHC I axis-induced autoimmune airway epithelial niche modulated by stemness-exhausted aged club cells may be potentiated by monocytes via IFN-γ stimulator IL18 and could facilitate COPD development via cytotoxic damage of airway epithelia. Under smoking conditions, enhanced intercellular communications between macrophages and endothelial cells depending on both ligands–receptors interactions and bioactivated sphingolipids metabolites impair pulmonary endothelial barriers, which could exacerbate inflammatory destruction of alveolar epithelia with a concomitant increased capacity for antigen presentation via MHC class II of innate immune cells including monocytes, DCs, macrophages, and neutrophils

In the current study, we presented a human COPD lung atlas generated via the analysis of a scRNA-seq dataset of lung tissue samples from 3 COPD patients, 3 young healthy individuals, and 3 elderly healthy individuals with different smoking histories. Based on a cell-type prioritization analysis, we first identified monocytes, club cells, and macrophages as the top-ranked COPD-, aging-, and smoking-associated cells and then clarified 3 condition-specific system-level malfunctions that could all be reflected by monocytes. Due to the control of the impact of aging and smoking, we defined a “COPD core” pathology characterized by a monocyte-derived detrimental inflammatory signaling pathway targeting alveolar epithelial cells. All the condition-specific system-level malfunctions associating to COPD were integrated into a new COPD pathological model (Fig. 8), wherein aging and smoking facilitate COPD via the autoimmune airway epithelial niche regulated by stemness-exhausted club cells and elevated intercellular communications between macrophages and endothelial cells, respectively. After combining our dataset with publicly available datasets, we finally confirmed our findings by combined analysis.

Recently, the increased utilization of scRNA-seq outlined the transcriptional heterogeneity of COPD [17–22], providing a brand new perspective for probing COPD pathobiology at a high-resolution. Several studies identified specific subpopulations of cells (especially among alveolar epithelial cells) from COPD lungs [18–20], revealing cell-type-specific disease mechanisms. Some presented signaling networks across multiple cell types (usually between structural and immune cells) [18–21], depicting the changes in cellular crosstalk during COPD development. Intriguingly, these findings, some of which were consistent with what we observed in our dataset [17, 21, 22], generally concentrated in the loss of alveolar homeostasis and chronic inflammation from the heterogenous biologic processes involved in COPD pathogenesis. Herein, to further disentangle COPD heterogeneity while taking aging and smoking into consideration, we collected samples from age-stratified donors with different smoking histories. Using analytic strategy based upon condition-specific cell-type prioritization and system-level malfunctions integration, we took a step forward in clarifying “COPD core” pathologies as well as the independent contributions of aging and smoking.

The importance of monocytes to pulmonary pathology has not been realized until recent years with the insight that beyond circulating precursors of tissue-resident macrophages and APCs involved in steady-state lung surveillance [45], they also serve as potent effector cells in numerous lung diseases [46–49]. Here, we identified the top-prioritized association of monocytes with COPD and their greatest contribution to COPD heritability among the various cell types analysed. Our further investigations demonstrated that monocytes under different conditions (including COPD, aging, and smoking) showed distinguishing immune statuses that were corresponding to those on systemic level while they also orchestrated “COPD core” pathogenesis, with different immunological roles under the conditions of aging and smoking. Therefore, our findings to some degree revealed the systemic inflammatory nature of COPD as well as the unappreciated potential of monocytes as a biomarker for assessing the pathological progression of COPD and a therapeutic target for COPD.

As a well-known heterogeneous and multifunctional source of progenitors for airway epithelial repair [50], club cells were confirmed to tridirectionally differentiate into AT1s, AT2s, and ciliated cells in our study. A distinct subpopulation of club cells marked by high expression of the MHC class I molecule H2-K1, which is very likely to be the counterpart of our autoimmune-prone sub-cluster of club cells, was recently described in the distal airway of adult murine lungs. When injured, this mobilizable and alveolar regeneration-promotive H2-K1+ subpopulation of club cells shows impaired self-renewal and features of senescence, limiting complete repair [51]. Our findings that the autoimmune-prone sub-cluster with distinctive stemness exhaustion was dramatically enriched in both COPD and aged lungs, complemented the role of the H2-K1 + subpopulation during aging and were fitted well with the classic view that an age-related decline in stem cell capacity for tissue repair can contribute to the development of age-associated diseases [52]. In addition, CCIs analysis revealed the strongest enhanced interactions of the top-prioritized aging-associated club cells with other cell types in aged lungs, reflecting the complex influence of aging on club cells and also putting emphasis on their modulatory effect on the microenvironment in aged lungs that increases susceptibility to COPD.

In our data, the correlation between mitochondrial gene expression and sphingolipid metabolism in macrophages may be attributed to the mitochondrial localization of serine palmitoyltransferase and ceramide synthase (CerS), which are encoded by *SPTLC2* and *CERS2,* respectively. They preferentially catalyze the de novo synthesis of long-chain ceramides, which directly influence mitochondrial gene expression after being generated in mitochondria and tend to be more dependent on vesicle-mediated transmembrane transportation than short-chain ceramides [53]. Besides, serine, the raw material for the de novo synthesis of ceramides in mitochondria and ER, was also found to influence mitochondrial dynamics and function [54]. Notably, both enhanced mitochondrial gene expression and significant changes in lipid metabolism have been confirmed in alveolar macrophages (AMs) from COPD patients [21]. As a cellular adaptation to elevated metabolic activity, the profound alterations in mitochondrial gene expression shared by macrophages from both smoking and COPD lungs may be indispensable to the survival of these reprogrammed pathogenic macrophages under high pathological stress, which partly explains the persistent pulmonary inflammation even after smoking cessation [55].

There are certainly some limitations of our study, including the sample collection based on non-tumorous adjacent lung tissues, the limited number of subjects from a single centre, and the in silico analysis of single-cell transcriptomic data only. Nonetheless, through our integrated analysis of our dataset combined with publicly available datasets from Adams et al*.* [22] and Li et al*.* [17], we extended our findings to groups with both sexes, different ages, smoking histories and genetic and clinical backgrounds. The remaining part of the CCIs in COPD lung tissues should be explored in the near future to expand and complete the connectomic landscape of “COPD core” pathogenesis with a monocyte centrality. Furthermore, in vitro and in vivo studies are needed to test the potential of monocytes as biomarker and therapeutic target for COPD.

## Conclusions

We identified monocytes, club cells, and macrophages as the top-prioritized COPD-, aging-, and smoking-associated cell types, respectively, from which we further characterized system-level malfunctions. Interestingly, we found condition-specific malfunctions could be distinguished by the immunological statuses of monocytes. In the end, we proposed an integrative COPD pathological model centred on a previously unrecognized pro-inflammatory signalling pathway from monocytes to alveolar epithelial cells, while aging and smoking facilitate the development of COPD via an autoimmune airway epithelial niche modulated by stemness-exhausted club cells and the imbalance of sphingolipids rheostat induced by macrophages, respectively. Our study provides a clarified view of COPD pathogenesis and demonstrates the potential utility of monocytes in COPD diagnosis and treatment.

## Supplementary Information

**Additional file 1. **Supplementary Methods.

**Additional file 2: Figures S1–S6.**

**Additional file 3: Dataset 1.** Demographics and clinical data of the 9 patients included in this study.

**Additional file 4: Dataset 2.** Results of differential gene expression analysis within each cell type under the conditions of aging, COPD, and smoking, respectively.

**Additional file 5: Dataset 3.** Lists of markers in 3 sub-clusters of monocytes, namely CD14+ classical monocytes, CD14+ intermediate monocytes, and CD16+ non-classical monocytes, related to Fig. S3B–S3D. The resulting sub-cluster markers were identified by the seurat FindAllMarkers, including the p-value, the average log2(fold change) of the gene in the sub-cluster compared to all other sub-clusters, the percent of cells expressing the gene in the sub-cluster (pct.1), the percent of cells expressing the gene in all other sub-clusters (pct.2), and the adjusted p-value.

**Additional file 6: Dataset 4.** Lists for the up-regulated COPD-associated DEGs in monocytes mentioned in Fig. S3I.

**Additional file 7: Dataset 5.** Genes dynamically expressed during CD14+ classical monocyte-to-CD14+ intermediate monocyte differentiation, related to Fig. S3M and S3N, in which the top 250 genes based on wald statistics were differentiation driver genes.

**Additional file 8: Dataset 6.** Gene lists for all co-expression gene modules of AT2s detected by WGCNA analysis shown in Fig. 4F.

**Additional file 9: Dataset 7.** Genes dynamically expressed during AT2-to-AT1 differentiation, related to Fig. S4D and S4G, in which the top 500 genes based on wald statistics were differentiation driver genes and those pro-inflammatory and/or pro-apoptotic ones were highlighted in red.

**Additional file 10: Dataset 8.** Lists of markers in 2 sub-clusters of club cells, namely autoimmune-prone sub-cluster of club cells and mix sub-cluster of club cells, related to Fig. 5D and Fig. S5M. The resulting sub-cluster markers were identified by the seurat FindAllMarkers, including the p-value, the average log2(fold change) of the gene in the sub-cluster compared to all other sub-clusters, the percent of cells expressing the gene in the sub-cluster (pct.1), the percent of cells expressing the gene in all other sub-clusters (pct.2), and the adjusted p-value.

**Additional file 11: Dataset 9.** Genes dynamically expressed during club cell-to-AT1, club cell-to-AT2s, and club cell-to-ciliated cell differentiation, related to Fig. 5F, in which the top 100 genes based on wald statistics were differentiation driver genes.

**Additional file 12: Dataset 10.** Lists for genes expressed in endothelial cells which are strongly correlated with genes from macrophages (Spearman correlation coefficient ≥ 0.8 or ≤ -0.8), related to Fig. 6H. Lists for genes expressed in macrophages which are strongly correlated with the 8 dysregulated sphingolipid metabolic enzyme genes in macrophages (Spearman correlation coefficient ≥ 0.8 or ≤ -0.8), related to Fig. 6H.

**Additional file 13: Dataset 11.** Demographics and clinical data of the 9 patients from Adams’ study integrated by us for validation, related to Fig. S3P, S3Q and Fig. 7.

## Data Availability

Some results of this study have been preprinted in bioRxiv (https://doi.org/10.1101/2021.02.23.432590). The datasets and source code of scRNA-seq analysis are available at GEO (GSE171541) and GitHub (https://github.com/wyy-linlab/ScRNASeq-COPD), respectively.

## Abbreviations

AMs: Alveolar macrophages
APCs: Antigen-presenting cells
ASAH1: *N*-Acylsphingosine amidohydrolase 1
AT1s: Alveolar type 1 cells
AT2s: Alveolar type 2 cells
AZGP1: Alpha-2-glycoprotein 1, zinc-binding
B2M: β2-Microglobulin
BMDMs: Bone-marrow-derived macrophages
BST2: Bone marrow stromal cell antigen 2
CCIs: Cell–cell interactions
CCL20: C–C motif chemokine ligand 20
CCL4: C–C motif chemokine ligand 4
CCRL2: C–C motif chemokine receptor like 2
CD47: CD47 molecule
CD55: CD55 molecule (Cromer blood group)
CD74: CD74 molecule
CEACAM1: Carcinoembryonic antigen-related cell adhesion molecule 1
CERK: Ceramide kinase
CERS2: Ceramide synthase 2
CERS5: Ceramide synthase 5
COPD: Chronic obstructive pulmonary disease
CRNDE: Colorectal neoplasia differentially expressed
CRTAM: Cytotoxic and regulatory T cell molecule
CSE: Cigarette smoke extract
CXCR4: C–X–C motif chemokine receptor 4
DEGs: Differentially expressed genes
EIF3E: Eukaryotic translation initiation factor 3 subunit E
ER: Endoplasmic reticulum
EREG: Epiregulin
FAS: Fas cell surface death receptor
FGF: Fibroblast growth factor
FGFR2: Fibroblast growth factor receptor 2
FLNA: Filamin A
GO: Gene Ontology
GSEA: Gene set enrichment analysis
GSVA: Gene set variation analysis
GWAS: Genome-wide association study
H2-K1: Histocompatibility 2, K1, K region
HAVCR2: Hepatitis A virus cellular receptor 2
HNRNPM: Heterogeneous nuclear ribonucleoprotein M
ICAM1: Intercellular adhesion molecule 1
IFNAR1: Interferon alpha and beta receptor subunit 1
IFNAR2: Interferon alpha and beta receptor subunit 2
IFNGR1: Interferon gamma receptor 1
IFNGR2: Interferon gamma receptor 2
IFN-γ: Interferon gamma
IGFBP7: Insulin like growth factor binding protein 7
IL-18: Interleukin-18
IL1B: Interleukin 1 beta
IL7R: Interleukin 7 receptor
IRE1: Endoplasmic reticulum to nucleus signaling 1
KLF4: Kruppel like factor 4
MAP2K3: Mitogen-activated protein kinase 3
MAPK: Mitogen-activated protein kinases
MHC class I: Major histocompatibility complex class I
MHC class II: Major histocompatibility complex class II
MPS: Mononuclear phagocyte system
NF-κB: Nuclear factor-kappa B
NKCs: Natural killer cells
NOTCH2: Notch receptor 2
PDGF: Platelet derived growth factor
REL: REL proto-oncogene, NF-kB subunit
scRNA-seq: Single-cell RNA sequencing
SGPP1: Sphingosine-1-phosphate phosphatase 1
SMPD4: Sphingomyelin phosphodiesterase 4
SNRPG: Small nuclear ribonucleoprotein polypeptide G
SOX2: SRY-box transcription factor 2
SPTLC1: Serine palmitoyltransferase long chain base subunit 1
SPTLC2: Serine palmitoyltransferase long chain base subunit 2
Th1: T helper type 1
TIE1: Tyrosine kinase with immunoglobulin like and EGF like domains 1
TIMP2: TIMP metallopeptidase inhibitor 2
TMEM45A: Transmembrane protein 45A
TNF: Tumor necrosis factor
TNFRSF1A: TNF receptor superfamily member 1A
TWSG1: Twisted gastrulation BMP signaling modulator 1
UMAP: Uniform manifold approximation and projection
UMI: Unique molecular identifier
USP47: Ubiquitin specific peptidase 47
USP7: Ubiquitin specific peptidase 7
WGCNA: Weighted gene coexpression network analysis
YAP1: Yes1 associated transcriptional regulator
