# Supplementary material for: Single-cell transcriptomics highlights immunological dysregulations of monocytes in the pathobiology of COPD
Source: Respir Res. 2022 Dec 20;23:367. doi: 10.1186/s12931-022-02293-2 (PMC9764587; doi:10.1186/s12931-022-02293-2)
Supplement: Supplementary file 5 — Additional file 5: Dataset 3. Lists of markers in 3 sub-clusters of monocytes, namely CD14+ classical monocytes, CD14+ intermediate monocytes, and CD16+ non-classical monocytes, related to Fig. S3B–S3D. The resulting sub-cluster markers were identified by the seurat FindAllMarkers, including the p-value, the average log2(fold change) of the gene in the sub-cluster compared to all other sub-clusters, the percent of cells expressing the gene in the sub-cluster (pct.1), the percent of cells expressing the gene in all other sub-clusters (pct.2), and the adjusted p-value. [file 12931_2022_2293_MOESM5_ESM.pdf]

Supplementary Methods

Generation of single-cell suspensions from lung tissues

Upon arrival to the laboratory, lung tissue (up to 20 × 20 × 20 mm) was washed with icy sterile PBS and was processed to prepare a single-cell suspension. Briefly, lung tissue was cut into pieces and incubated with 2 mg/ml collagenase I, 2 mg/ml collagenase IV and 0.2 mg/ml DNase I (Roche) in RPMI 1640 for 40 min at 37°C with 150 rpm shaking. The resulting cell suspension was filtered through a 70-µm sterile nylon strainer and centrifuged at 400 g for 5 min at 4°C. The cell pellet was resuspended in 1 ml RBC lysis buffer (0.15 mol/l NH4Cl, 10 mmol/l KHCO3, 0.1 mmol/l EDTA) and lysed for 10 min at room temperature. Cells were centrifuged at 400 g for 5 min at 4°C. The cell pellet was resuspended in 400 µl PBS for cell sorting by Countstar (IC1000).

Single-cell RNA sequencing cell capture and cDNA synthesis

Using BD Rhapsody™ Cartridge Reagent Kit (BD, 633731) and BD Rhapsody™ Cartridge Kit (BD, 633733), the cell suspension (300-600 living cells per microliter determined by Count Star) was loaded onto the Rhapsody™ Cartridge (BD) to generate single-cell magnetic beads in the microwells according to the manufacturer's protocol. In short, single cells were suspended in sample buffer (BD). About 18,000 cells were added to each channel, and the target cell will be recovered was estimated to be about 9,000 cells. Captured cells were lysed and the released RNA were

barcoded through reverse transcription in individual microwells. Reverse transcription was performed on a ThermoMixer® C (Eppendorf) at 1,200 rpm and 37°C for 45 minutes. The cDNA was generated and then amplified, and quality assessed using an Agilent 4200 (performed by CapitalBio Technology, Beijing). Single-cell RNA sequencing (scRNA-seq) library preparation according to the manufacture's introduction, scRNA-seq libraries were constructed using BD Rhapsody™ WTA Amplification Kit (BD, 633801). The libraries were finally sequenced using an Illumina Novaseq6000 sequencer with a sequencing depth of at least 50,000 reads per cell with pair-end 150 bp (PE150) reading strategy (performed by CapitalBio Technology, Beijing).

Analysis of scRNA-seq data

The BD Rhapsody analysis pipeline was used to process sequencing data (.fastq files) and the reference genome was GENCODE v29 [1]. The scRNA-seq expression matrix was processed with the R package Seurat (version 3.1.5) [2]. The cells were removed that had either fewer than 301 expressed genes or over 30% UMIs originating from mitochondrial. UMI counts were normalized with a scale factor of 10,000 UMIs per cell and subsequently log transformed. Three thousand highly variable Genes (HVGs) of each data were identified by the 'FindVariableFeatures' function. Integration of 9 individual dataset was performed to correct batch effect. Expression matrix was summarized by the top 14 principal components. Visualization of transcriptomic

profiles was conducted by uniform manifold approximation and projection (UMAP). The Louvain modularity optimization algorithm was applied to iteratively group cells together into clusters for cell type identification. Cell clusters were annotated to known biological cell types using canonical cell marker genes.

Differential gene expression analysis

To identify genes differentially expressed under three conditions (aging, smoking, and COPD) in each cell type, MAST (1.14.0) [3] was used to perform zero-inflated regression analysis by fitting a linear mixed model. We controlled both technical variation and individual variation by using a two-part hurdle model. The following model was fit with MAST:

zlm(~ COPD+ age + (1|individual) + smoking + percent.mt + nCount_RNA , sca, method='glmer', ebayes=FALSE)

Where nCount_RNA was the total number of molecules detected within a cell and percent.mt was mitochondria fraction. To identify genes differentially expressed due to the disease effect, likelihood ratio test was performed by comparing the model with and without the COPD variable. Multiple hypothesis testing was performed with Bonferroni and Holm corrections. For COPD/smoking variable, **genes** with absolute value of log2(fold change) > 0.14 (10% difference in **expression**) and FDR < 0.05 between conditions were selected as differentially expressed genes (DEGs). For aging variable, we treat aging as a continuous covariate while controlling for technical

variation and individual variation. Genes were considered as significantly differentially expressed with an FDR < 0.05 and an age coefficient > 0.2 or < -0.2.

Enrichment analysis of gene sets

Gene Ontology Gene enrichment was performed using the R package clusterProfiler (version 3.14.3) [4]. Enrichment in GO Biological process between DEGs of COPD of each cell type was analyzed using the compareCluster. Significantly enriched GO terms were simplified using simplify function remove highly similar terms (cutoff = 0.7) by retaining the most significant representative term. Enrichment in GO Biological process on DEGs of aging and smoking were analyzed as above. Enrichment analysis using custom genes sets was performed using the appropriate background lists (genes detected >10 % of cells in each cell type). Enrichment of COPD-associated DEGs at COPD GWAS risk genes[5] was performed using Fisher's exact test to determine cell types contributing to COPD heritability.

Downsampling analysis to identify condition-relevant cell types

To calculate the number of COPD-associated DEGs across cell types normalized by the number of cells in each cell type, we randomly drew 700 cells from each cell type before performing differential gene expression analysis. This analysis was repeated 10 times and the number of DEGs for each cell type were presented as boxplots. Aging-associated DEGs and smoking-associated DEGs were performed as above.

Cell type prioritization analysis

To delineate the cell type-specific responses to different biological conditions (aging, smoking, and COPD), cell type prioritization analysis in three conditions was performed using 'calculate_auc' function implemented in the Augur R package with default parameters (version 1.0.0) [6]. As input, we used the gene expression counts that were normalized with a scale factor of 10,000 UMIs per cell and subsequently natural log-transformed. Different conditions were analyzed separately. COPD and smoking cell type prioritization scores from augur analysis were AUC, and aging cell type prioritization score from augur analysis was CCC. The cell type prioritization score was displayed in heatmap.

Sub-clustering and identification of markers of sub-cluster

To identify sub-clusters within monocytes, we reanalyzed cells belonging to monocytes with a resolution of 0.2, and three sub-clusters of monocytes were identified: CD14+ classical monocytes, CD14+ intermediate monocytes and CD16+ non-classical monocytes. we further clustered our club cells with a resolution of 0.1 and generated 2 sub-clusters: mix sub-cluster, autoimmune-prone sub-cluster.

To distinguish sub-clusters within dendritic cells, we reanalyzed cells belonging to dendritic cells with a resolution of 0.2. According to the gene expression of each sub-cluster, we obtained five dendritic clusters, including IGSF2+ DC, TREM2+ DC,

myeloid DC1 (mDC1), myeloid DC2 (mDC2), plasmacytoid DC (pDC). To identify sub-clusters within macrophage, we reanalyzed cells belonging to macrophage with a resolution of 0.2, and two sub-clusters of macrophage were identified: one FABP4+ macrophage and one FABP4- macrophage.

We then applied the Seurat FindAllMarkers to generate cell type sub-cluster marker.

Gene set enrichment analysis (GSEA)

To determine whether differential expression genes within a given cell type were significantly enriched with meaningful biological processes, gene set enrichment analysis (GSEA) was done using the clusterProfiler R package (3.12.0) [4]. Only genes detected in >10% of cells of a given cell type were evaluated. All annotated gene sets were collected from the Molecular Signatures Database (MSigDB: C5: ontology gene sets). Gene set with a P-value less than 0.05 was considered as significantly enriched.

Trajectory analysis

To identify lineage trajectories, we used Slingshot (version 1.4.0) [7]. The cluster representing CD14+ classical monocytes, AT2s, and club cells were chosen as the root node separately to construct lineages of differentiation to other cell types based on PCA dimension reduction and estimate pseudotime of each cell along the lineages. After running slingshot, we used the tradeSeq package (version 1.4.0) [8] to find genes dynamically expressed during cell differentiation. For each HVG, we fit a general

additive model (GAM) using a negative binomial noise distribution to model the relationships between gene expression and pseudotime and tested for significant associations between expression and pseudotime using the associationTest. We picked out the top genes based on wald statistics and visualized their expression over developmental time with a heatmap.

Monocytes differentiation driver genes enrichment analysis

To identify lineage trajectories of monocytes sub-clusters, we performed trajectory inference analysis using Slingshot. The cluster representing CD14+ classical monocytes was chosen as the root node to construct lineages of differentiation to CD14+ intermediate monocytes. we fit a general additive model (GAM) using a negative binomial noise distribution to model the relationships between gene expression and pseudotime and tested for significant associations between expression and pseudotime using the associationTest. We ranked genes according to wald statistics (the gene with the largest waldStat is ranked the top) and picked out the top 250 genes based on wald statistics as monocytes differentiation driver genes. To investigate whether DEG sunder three conditions (COPD, aging, smoking) in monocytes are enriched in the pre-ranked 250 monocytes differentiation driver genes, we performed Kolmogorov-Smirnov (K-S) test on DEGs of monocytes and visualized with a kolmogorov-smirnov plot. The one-sided (greater) K-S test was used to determine the enrichment score. The nominal p-value estimates the statistical

significance of a single gene set's enrichment score, based on the null distribution generated from gene set permutation.

Cell-cell interactions (CCIs) analysis

To infer potentially relevant interactions between two cell populations, CCIs analysis was conducted with the CellPhoneDB software (version 2.0) [9]. For the COPD condition, normalized gene expression matrix of control and COPD patients was first corrected to remove variation due to smoking and aging using WGCNA R package function ‘empiricalBayesLM’. The same method is used to eliminate variation due to COPD and smoking in the aging condition and variation due to COPD and aging in the smoking condition. In CCIs analysis, COPD and control were analyzed separately. Only receptors and ligands detected in >10% of cells in each cell type from COPD were further evaluated, while a CCI was considered nonexistent if the ligand or the receptor was unmeasurable. Averaged expression of each ligand-receptor pair was analyzed between various cell types, and only those whose average expression value was in the top 75% quantile among the ligand-receptor pairs with p-value < 0.05 were used for the prediction of CCIs between any two cell types. The control group was analyzed as above. We quantified the degree of change in cell interactions in the COPD condition by the difference between the number of significant interaction ligand-receptor pairs in COPD and the number of significant interaction ligand-receptor pairs in control. The degree of changes in CCIs under aging (old vs. young

group) and smoking (active smoker vs. never-smoker group) conditions was analyzed as above.

Intercellular interaction network of ligands and receptors

To identify and illustrate alterations in the intercellular signaling network, iTALK (version 0.1.0) [10] (Corpus ID: 91802336) was employed to identify significant changes, i.e. gains or losses of interactions between groups. All ligands and receptors in analysis were collected from Chemical Classification and Information Database (CCID). Normalized cell-gene expression matrix that was corrected to remove variation due to smoking and aging using WGCNA R package function 'empiricalBayesLM' as input. For all COPD subject cells and each cell type, the expression value for each gene was averaged to create a single "sample" representative of a cell type of COPD. The ligand or receptor was considered expressed in monocytes if the averaged expression of monocytes was greater than 0.2, and the ligand or receptor was considered expressed in each cell type (except the monocytes) if the averaged expression per cell type was greater than 1.1. The number of ligand-receptor interactions between each two cell types was detected by 'FindLR' function in iTALK. The number of ligand-receptor interactions between each two different cell types of control was analyzed as above. The interaction alteration of each cell type between COPD and control was defined as follows: the difference between the number of interactions of the COPD group and the number of interactions

of the control group and then divided by the number of interactions of the control group. For visualization of interaction networks of iTALK cell-cell communication changes, colors in the interaction network correspond to the cell type, while the thickness of the lines is the changes of interaction pairs between cell types. To profile interaction alterations of ligand-receptor pair of monocytes with structural cells AT1/AT2 within COPD, we used ligand-receptor pairs from cell-cell interaction databases to analyze the interaction alteration between monocytes and structural cells based on the expression. Only differentially expressed ligands/receptors were further evaluated. The circosplot of interaction alterations using circlize R package (version 0.4.11) [11]. The interaction between monocytes and immune cells within COPD was analyzed as above.

Regulatory potential analysis

To speculate the regulatory potential of ligands in monocytes on differential expressed genes in interacting cells, we applied the NicheNet analysis using nichenetr R package (version 1.0.0) [12]. As input genes to infer the ligand activity score, we defined all DEG genes with FDR < 0.05 and log2fc > 0 within a given cell type in COPD condition. As background genes, we defined all genes that were not DEG within a given cell type in COPD conditions and detected in > 10% of cells. For ligand prioritization, we selected the genes with FDR< 0.05 and log2fc > 0.14 in COPD conditions were defined as large change potential_ligands within a given cell type.

Genes with FDR < 0.05 in COPD conditions were defined as potential_ligands within a given cell type.

Potential upstream regulators prediction

To predict potential upstream transcriptional regulators of the COPD-associated DEGs in AT1s, AT2s, endothelial cells, stromal cells, club cells, and ciliated cells, we performed enrichment analysis of DEGs using Metascape software [13]. TRRUST ontology categories with a p-value < 0.01, a minimum count of 3, and an enrichment factor > 1.5 in metascape were considered as significant DEG potential upstream regulators. The pathway annotation of upstream regulators was classified according to the pathway types in the KEGG database.

Weighted correlation network analysis (WGCNA)

To identify the co-expression gene modules of alveolar type 2 cells, a weight gene co-expression network analysis was performed using WGCNA R package (version 1.69) [14]. Expression was first corrected to remove variation due to smoking and aging using empiricalBayesLM. The appropriate soft-thresholding power was chosen based on a scale-free topology criterion and the weighted adjacency matrix was constructed using the soft-thresholding power. The relationship between one gene and all other ones in the analysis was incorporated, and the adjacency matrix was transformed into the topological matrix (TOM). The genes demonstrated hierarchical clustering according

to the TOM-based dissimilarity (1-TOM) measure, and highly interconnected genes were assigned to the same module. Then, the eigengenes of WGCNA modules were calculated to represent the overall gene expression profiles in each module and then were tested for statistical significance of association with the COPD. Hub genes are defined as genes with high correlation with the remaining genes in the Blue module.

Functional interaction analysis

To better demonstrate the core biological process in COPD-associated AT2s as well as their interrelationships, we used the STRING database [15] to construct a functional interaction network based on the genes which were enriched in more than 2 biological processes in the enrichment map showing the functional topologies among major biological process of AT2s WGCNA Blue module. Network type was physical complex and the edge thickness indicate the strength of data support. Active interaction source was from experiments only. The minimum required interaction score based upon confidence was set as 0.5.

AT2-to-AT1 differentiation driver genes enrichment analysis

To identify lineage trajectories of AT2s to AT1s, we performed trajectory inference analysis using Slingshot. The cluster representing AT2s was chosen as the root node to construct lineages of differentiation to AT1s. We ranked genes according to wald statistics (the gene with the largest waldStat is ranked the top) and picked out the top

500 genes based on wald statistics as AT2s-AT1s differentiation driver genes. To investigate whether COPD-, aging-, and smoking-associated DEGs of monocytes are enriched in the 500 AT2s-AT1s differentiation driver genes, we performed Kolmogorov-Smirnov (K-S) test on COPD-, aging-, and smoking-associated DEGs of monocytes and visualized with a kolmogorov-smirnov plot. The one-sided (greater) K-S test was used to determine the enrichment score. The nominal p-value estimates the statistical significance of a single gene set's enrichment score, based on the null distribution generated from gene set permutation.

Gene set variation analysis (GSVA)

To evaluate the activity of gene sets within a given cell type under different conditions, Gene set variation analysis (GSVA) was performed in the GSVA R package (version 1.34.0) [16]. All ontology gene sets were collected from the Molecular Signatures Database (MSigDB). Only genes detected in >10% of cells within a given cell type were further evaluated. As GSVA input, normalized gene expression matrix was first corrected to remove variation due to aging and smoking using WGCNA R package function 'empiricalBayesLM' in each cell type for the COPD condition; normalized gene expression matrix was first corrected to remove variation due to COPD and smoking using WGCNA function 'empiricalBayesLM' in each cell type for the aging condition; normalized gene expression matrix was first corrected to remove variation due to COPD and aging using WGCNA R package

function ‘empiricalBayesLM’ in each cell type for the smoking condition. Differential expression analysis with gene sets enrichment scores as if they were simple gene expression values using the ‘lmFit’ and ‘eBayes’ functions from the limma R package. Gene sets with adjust p-value (padjust) < 0.05 were selected as differentially activated gene sets.

Club differentiation driver genes functional enrichment analysis

To identify potential driver genes responsible for club differentiation from the club cells to the ciliated cells, AT1s, and AT2s, we performed trajectory analysis using Slingshot. Club cells was chosen as the root node to construct lineages of differentiation to ciliated cells, to AT1s, and to AT2s. For club cells to ciliated cells, we ranked genes according to wald statistics (the gene with the largest waldStat is ranked the top) and picked out the top 100 genes based on wald statistics as potential driver genes responsible for club cells differentiation from the club cells to the ciliated cells. Finally, we performed functional enrichment analysis on club cells differentiation driver genes using Metascape software. Club cell-to-AT1 and club cell-to-AT2 differentiation driver genes were identified as above.

Identification of macrophage subtypes

To identify macrophage subtype, we selected top 400 biological process terms of macrophage with largest standard deviation of GSVA enrichment score. K-means

clustering with k = 4 was performed on top 400 biological process GSVA enrichment score of macrophages, and 3 subtypes of macrophage were identified: k-means cluster1 and k-means cluster2 defined as subtype A, k-means cluster3 as subtype B and k-means cluster4 as subtype C.

Correlation analysis based on summarized expression profiles at the individual level

We calculated cell type-specific mean expression within each individual. As a result, each individual has an expression matrix with rows corresponding to genes and columns corresponding to cell types (15 columns in total). To estimate expression correlations between cell types across individuals, for example "A" gene in macrophages and "B" gene in endothelial cells, we actually performed Spearman correlation analysis between mean expression values of A genes of 9 individuals in macrophages and mean expression values of B genes of 9 individuals in endothelial cells. Highly correlated genes are defined as those with Spearman correlation coefficient ≥ 0.8 or ≤ -0.8.

Integration of publicly available scRNA-seq data

To assess the reliability of the three conditions (COPD, aging, and smoking)-associated DEGs in monocytes in our dataset, we collected COPD-associated DEGs in monocytes from the scRNA-seq dataset of Li and his colleagues [17]. Since they

neither set young controls nor analyzed smoking separately, both aging- and smoking-associated DEGs of monocytes were not available in their datasets but all mixed with COPD-associated DEGs. GO enrichment analysis was performed across our three conditions (COPD, aging, and smoking)-associated DEGs of monocytes and COPD-associated DEGs of Li and his colleagues' via Metascape database [13]. GO biological process terms with moderate p-value were selected to eliminate some biological processes too broad to reflect the specific pathological role and displayed in a heatmap. To further assess the reliability of the three conditions (COPD, aging, and smoking)-associated DEGs in CD14+ monocytes in our dataset, we collected COPD-associated DEGs in CD14+ monocytes from the scRNA-seq dataset of Adams and his colleagues [18]. GO enrichment analysis across these multiple DEGs were performed and visualized as above.

Data from GEO accession number GSE136831, representing scRNA-seq raw count data from 6 control lung homogenates and 3 transplant stage COPD lungs, were used. Samples' clinical information are given in Dataset 7.xlsx.

To validate the reproducibility of our study, all cells of 9 samples in our study and those of 9 samples in Adams' study were combined as the "9 + 9 dataset".

To identify genes differentially expressed under three conditions (aging, smoking, and COPD) within each cell type of the 9 + 9 dataset, MAST was used to perform zero-inflated regression analysis by fitting a linear mixed model. Considering the batch effect of the 9 + 9 dataset, our 9 samples were defined as batch 1 data, while Adams'

9 samples were defined as batch 2 data. We controlled both technical variation and individual variation by using a two-part hurdle model. The following model was fit with MAST:

zlm(~ COPD+ age + (1|individual) + smoking + batch + sex+ percent.mt + nCount_RNA , sca, method='glmer', ebayes=FALSE)

For COPD/smoking variable, genes with absolute value of log2(fold change) > 0.14 (10% difference in expression) and FDR < 0.05 between conditions were selected as differentially expressed genes (DEGs). For aging variable, we treat aging as a continuous covariate while controlling for technical variation and individual variation. Genes were considered as significantly differentially expressed with an FDR < 0.05 and an age coefficient > 0.2 or < -0.2.

To calculate the number of COPD-associated DEGs across cell types normalized by the number of cells in each cell type of the 9 + 9 dataset, we randomly drew 2000 cells from each cell type before performing differential gene expression analysis. This analysis was repeated 10 times and the number of DEGs for each cell type were presented as boxplots. Aging-associated DEGs and smoking-associated DEGs were performed as above. Since no neutrophil cells were annotated as neutrophils in Adams' data, neutrophils were not included in downsampling analysis of the 9 + 9 dataset.

To verify the reliability of GSEA analysis results in our study, we applied the differential expression analysis results of the 9 + 9 dataset to conduct GSEA analysis,

and the analysis process was as described in the previous gene set enrichment analysis.

Preparation of aqueous cigarette smoke extract (CSE)

Huangshan Brand cigarettes were manufactured by China Tobacco Anhui Industrial Co., Ltd. Tar, nicotine, and carbon monoxide contents of Huangshan Brand cigarettes were 10 mg/cigarette, 0.9 mg/cigarette, and 11 mg/cigarette, respectively. CSE was prepared by bubbling smoke from 1 cigarette into 10 ml of RPMI medium 1640 (Gibco, Burlington, ON, USA) at a rate of 1 cigarette per 2 minutes, as described previously [19]. The pH of CSE was adjusted to 7.2-7.4. After being filtered through a sterile 0.22 μm filter (Merck&Millipore,Darmstadt, Germany), some CSE preparation was diluted 7 times with RPMI medium 1640 and used to monitor the absorbance at 320 nm (optical density of 0.67 ± 0.01). CSE was freshly prepared for each experiment and diluted with culture medium immediately before use. Control medium was prepared by bubbling air through 10 ml of culture medium, adjusting pH to 7.4, and sterile filtering as described for CSE preparation.

Generation and treatment of bone marrow-derived macrophages (BMDMs) from mouse

All animal experiments were approved by the Institutional Animal Care and Use Committee at Nanjing Medical University. Male C57BL/6 mice of 8 weeks were

purchased from the National Resource Center for Mutant Mice Model Animal Research Center of Nanjing University. GM-CSF derived alveolar-like macrophages were generated from bone marrow of unchallenged C57BL/6 mice as previously described [20]. Briefly, mouse was euthanized and its femurs and tibiae were isolated in the biological safety cabinet. Cut the end of each bone.

Insert the tip of a $26^{1/2}$ gauge (G) needle (BD, NJ, USA) into the end of a bone piece and flush out the bone marrow cells with 1 ml Hank's Balanced Salt Solution with 5% fetal calf serum (Gibco) (HBSS-FCS) into a 100 mm x 15 mm sterile dish (Corning, NY, USA). Transfer the born marrow suspension to a sterile 50 ml conical tube (Corning). Spin down the bone marrow cells at 314 g for 5 min at 4 °C. Aspirate the supernatant and loosen the pellet by adding 10 ml of red blood cell (RBC) lysis buffer (Beyotiome, Shanghai, China). Vortex to resuspend the cells and incubate them in RBC lysis buffer at room temperature for 5 min. Add 10 ml of HBSS-FCS then spin cells at 314 g for 5 min and prepare born marrow culture (BMC) media by adding 10% FCS, 20 mM HEPES (Gibco), 1x nonessential amino acid (Solarbio, Beijing, China), 55 μM 2-mercaptoethanol (Sigma Aldrich, St. Louis, MO, USA), 50 U/ml penicillin and streptomycin (Solarbio), 1 mM sodium pyruvate (Solarbio), and 2 mM L-glutamine (Solarbio) to RPMI medium 1640 (Gibco). Aspirate the supernatant and resuspend cells at 4×10^6 cells/ml in BMC media. Add 500 μl of the 4×10^6 cells/ml bone marrow cells to 9.5 ml of BMC media containing 200 ng of recombinant mouse GM-CSF (PeproTech, Rocky Hill, NJ, USA), plant bone marrow cells in a new sterile

100 mm x 15 mm Petri dish for the final concentration of 2 x 10^5 cells/ml and culture them at 37°C in a humidified 5% CO_2 atmosphere. On day 3, add 10 ml of fresh BMC media containing 200 ng of GM-CSF to the dish of cells. On day 6, carefully remove 10 ml of media into a sterile 50 ml conical tube, spin down at 314 g for 5 min, and discard the supernatant. Resuspend the cell pellet in 10 ml of fresh BMC media containing 20 ng/ml of GM-CSF, gently add to the original cell culture dish for a total volume of 20 ml. On day 7, harvest the non-adherent cells in a sterile 50 ml conical tube. Spin down at 314 g for 5 min, discard the supernatant and resuspend the cell pellet at 1 x 10^6 cells/ml in BMC media with CSE at desired concentration or same volume of control medium. Culture cells in new 60 mm x 15 mm sterile dishes (Corning) at 37°C in a humidified 5% CO_2 atmosphere for another 24 h before final collection.

RNA extraction and RT-PCR assay

Total RNA was extracted from macrophages using Trizol reagent (Invitrogen, Grand Island, NY, USA) as previously described [21]. RNA was reverse-transcribed into cDNA with HiScript II Q RT SuperMix for qPCR (Vazyme, Nanjing, Jiangsu, China). Quantification RT-PCR was performed using ChamQTM Universal SYBR qPCR Master Mix (Vazyme) and Step One PlusTM Real-Time PCR System (Applied Biosystems, Foster City, CA, USA). Primers were manufactured by Genscript (Nanjing, Jiangsu, China). *Arpppo* was used as internal standards for mRNAs. Primer

sequences were designed to be intron-spanning and were as follows (5′-3′): *Arpppo* (forward: GAAACTGCTGCCTCACATCCG, reverse: GCTGGCACAGTGACCTCACACG); mouse *Sptlc1* (forward: AGGGCATCGCATTGACTC, reverse: GGTCTCTGAGTAGTTCCGTCCGCAG); mouse *Sptlc2* (forward: GTTGCCTGATTCTGAGTG, reverse: TTCTTCCAGGGTCTTCTT); mouse *Asah1* (forward: AAAGTCTTCTCACCTGGGTC, reverse: ACGGAACTGGTCCTCTAT); mouse *Cers2* (forward: GCCCAAGCAGGTGGAGGTA, reverse: TGACAGCCATGCCAGCAA); mouse *Cers5* (forward: CAGGACAAGCCTCCAACG, reverse: CGGGTATCCCAGAACCAA); mouse *Sgpp1* (forward: CGGGTATCCCAGAACCAA, reverse: AATCTCAGCCGTGTCTCC); mouse *Cerk* (forward: CCATCAATGCCACCAACA, reverse: GACTCGATAAACTTCAACGAAA); mouse *Smpd4* (forward: GCAGTGCCTCGCTTTGTC, reverse: TGCCCTGAGCCTGTTTCT).

Statistical analysis

For the *in vitro* experiment related to Fig. 7E, at least three independent experiments were performed. Comparisons were performed using the Student’s t test between two groups. Results were presented as means ± SEM. A p-value < 0.05 was considered statistically significant.

References

1 Harrow, J. *et al.* GENCODE: the reference human genome annotation for The ENCODE Project. *Genome Res* **22**, 1760-1774, doi:10.1101/gr.135350.111 (2012).

2 Stuart, T. *et al.* Comprehensive Integration of Single-Cell Data. *Cell* **177**, 1888-1902 e1821, doi:10.1016/j.cell.2019.05.031 (2019).

3 Finak, G. *et al.* MAST: a flexible statistical framework for assessing transcriptional changes and characterizing heterogeneity in single-cell RNA sequencing data. *Genome Biol* **16**, 278, doi:10.1186/s13059-015-0844-5 (2015).

4 Yu, G., Wang, L. G., Han, Y. & He, Q. Y. clusterProfiler: an R package for comparing biological themes among gene clusters. *OMICS* **16**, 284-287, doi:10.1089/omi.2011.0118 (2012).

5 Sakornsakolpat, P. *et al.* Genetic landscape of chronic obstructive pulmonary disease identifies heterogeneous cell-type and phenotype associations. *Nat Genet* **51**, 494-505, doi:10.1038/s41588-018-0342-2 (2019).

6 Skinnider, M. A. *et al.* Cell type prioritization in single-cell data. *Nat Biotechnol* **39**, 30-34, doi:10.1038/s41587-020-0605-1 (2021).

7 Street, K. *et al.* Slingshot: cell lineage and pseudotime inference for single-cell transcriptomics. *BMC Genomics* **19**, 477, doi:10.1186/s12864-018-4772-0 (2018).

8 Van den Berge, K. *et al.* Trajectory-based differential expression analysis for single-cell sequencing data. *Nat Commun* **11**, 1201, doi:10.1038/s41467-020-14766-3 (2020).

9 Efremova, M., Vento-Tormo, M., Teichmann, S. A. & Vento-Tormo, R. CellPhoneDB: inferring cell-cell communication from combined expression of multi-subunit ligand-receptor complexes. *Nat Protoc* **15**, 1484-1506, doi:10.1038/s41596-020-0292-x (2020).

10 Yuanxin Wang, R. W., Shaojun Zhang, Shumei Song, Changying Jiang, Guangchun Han, Michael Wang, Jaffer Ajani, Andy Futreal, Linghua Wang. iTALK: an R Package to Characterize and Illustrate Intercellular Communication. *biorxiv preprint*, doi:10.1101/507871 (2019).

11 Gu, Z., Gu, L., Eils, R., Schlesner, M. & Brors, B. circlize Implements and enhances circular visualization in R. *Bioinformatics* **30**, 2811-2812, doi:10.1093/bioinformatics/btu393 (2014).

12 Browaeys, R., Saelens, W. & Saeys, Y. NicheNet: modeling intercellular communication by linking ligands to target genes. *Nat Methods* **17**, 159-162, doi:10.1038/s41592-019-0667-5 (2020).

13 Zhou, Y. *et al.* Metascape provides a biologist-oriented resource for the analysis of systems-level datasets. *Nat Commun* **10**, 1523, doi:10.1038/s41467-019-09234-6 (2019).

14 Langfelder, P. & Horvath, S. WGCNA: an R package for weighted correlation network analysis. *BMC Bioinformatics* **9**, 559, doi:10.1186/1471-2105-9-559 (2008).

15 Szklarczyk, D. *et al.* The STRING database in 2021: customizable protein-protein networks, and functional characterization of user-uploaded gene/measurement sets. *Nucleic Acids Res* **49**, D605-D612, doi:10.1093/nar/gkaa1074 (2021).

16 Hanzelmann, S., Castelo, R. & Guinney, J. GSVA: gene set variation analysis for microarray and RNA-seq data. *BMC Bioinformatics* **14**, 7, doi:10.1186/1471-2105-14-7 (2013).

17 Li, X. *et al.* Single cell RNA sequencing identifies IGFBP5 and QKI as ciliated epithelial cell genes associated with severe COPD. *Respir Res* **22**, 100, doi:10.1186/s12931-021-01675-2 (2021).

18 Adams, T. S. *et al.* Single-cell RNA-seq reveals ectopic and aberrant lung-resident cell populations in idiopathic pulmonary fibrosis. *Sci Adv* **6**, eaba1983, doi:10.1126/sciadv.aba1983 (2020).

19 Yang, S. R. *et al.* RelB is differentially regulated by IkappaB Kinase-alpha in B cells and mouse lung by cigarette smoke. *Am J Respir Cell Mol Biol* **40**, 147-158, doi:10.1165/rcmb.2008-0207OC (2009).

20 Dong, Y. *et al.* Generation and Identification of GM-CSF Derived Alveolar-like Macrophages and Dendritic Cells From Mouse Bone Marrow. *J Vis Exp*, doi:10.3791/54194 (2016).

21 Huang, Q. *et al.* Glucolipotoxicity-Inhibited miR-299-5p Regulates Pancreatic beta-Cell Function and Survival. *Diabetes* **67**, 2280-2292, doi:10.2337/db18-0223 (2018).
