# Supplementary material for: Single-cell transcriptomics highlights immunological dysregulations of monocytes in the pathobiology of COPD
Source: Respir Res. 2022 Dec 20;23:367. doi: 10.1186/s12931-022-02293-2 (PMC9764587; doi:10.1186/s12931-022-02293-2)
Supplement: Supplementary file 6 — Additional file 6: Dataset 4. Lists for the up-regulated COPD-associated DEGs in monocytes mentioned in Fig. S3I. [file 12931_2022_2293_MOESM6_ESM.pdf]

Supplementary Figures

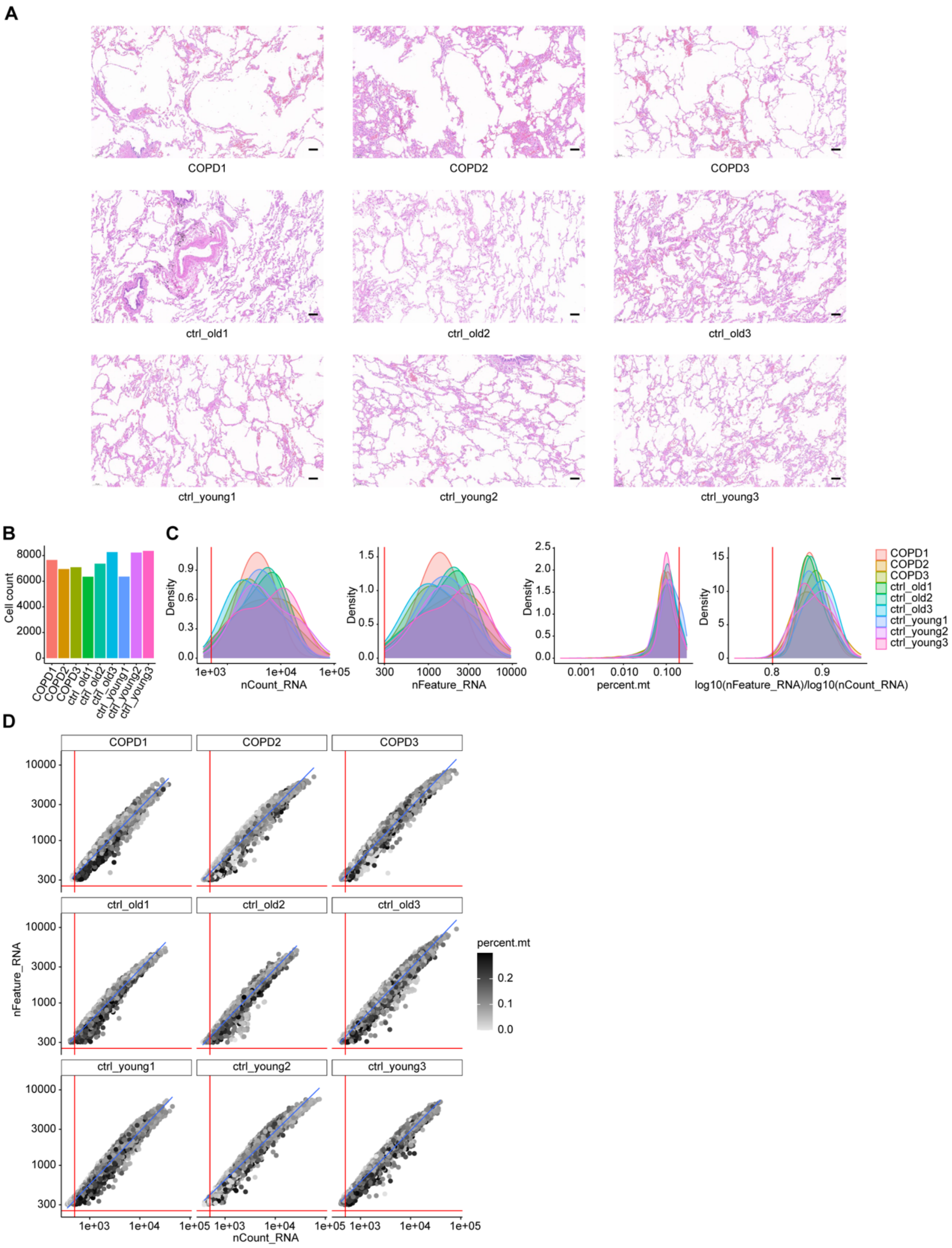

Figure S1.

(**A**) Hematoxylin-eosin (H&E) staining of lung tissues from all the subjects. scale bar = 100 μm.

(**B**) Bar plot showing the number of cell count per sample.

(**C**) From left to right are:

Density plot showing the number of RNAs per cell. Red line: 500.

Density plot showing the number of genes detected per cell. Red line: 300.

Density plot showing the percent of mitochondrial reads detected per cell. Red line: 0.2.

Density plot showing the ratio of log10(the number of genes) versus log10(the number of RNAs per cell).

(**D**) Scatter plot showing the number of genes versus the number of UMIs and was colored by the percent of mitochondrial reads. The color of the dot indicates the high (black) or the low (white) percent of mitochondrial reads. X intercept red line: 500; Y intercept red line 250.

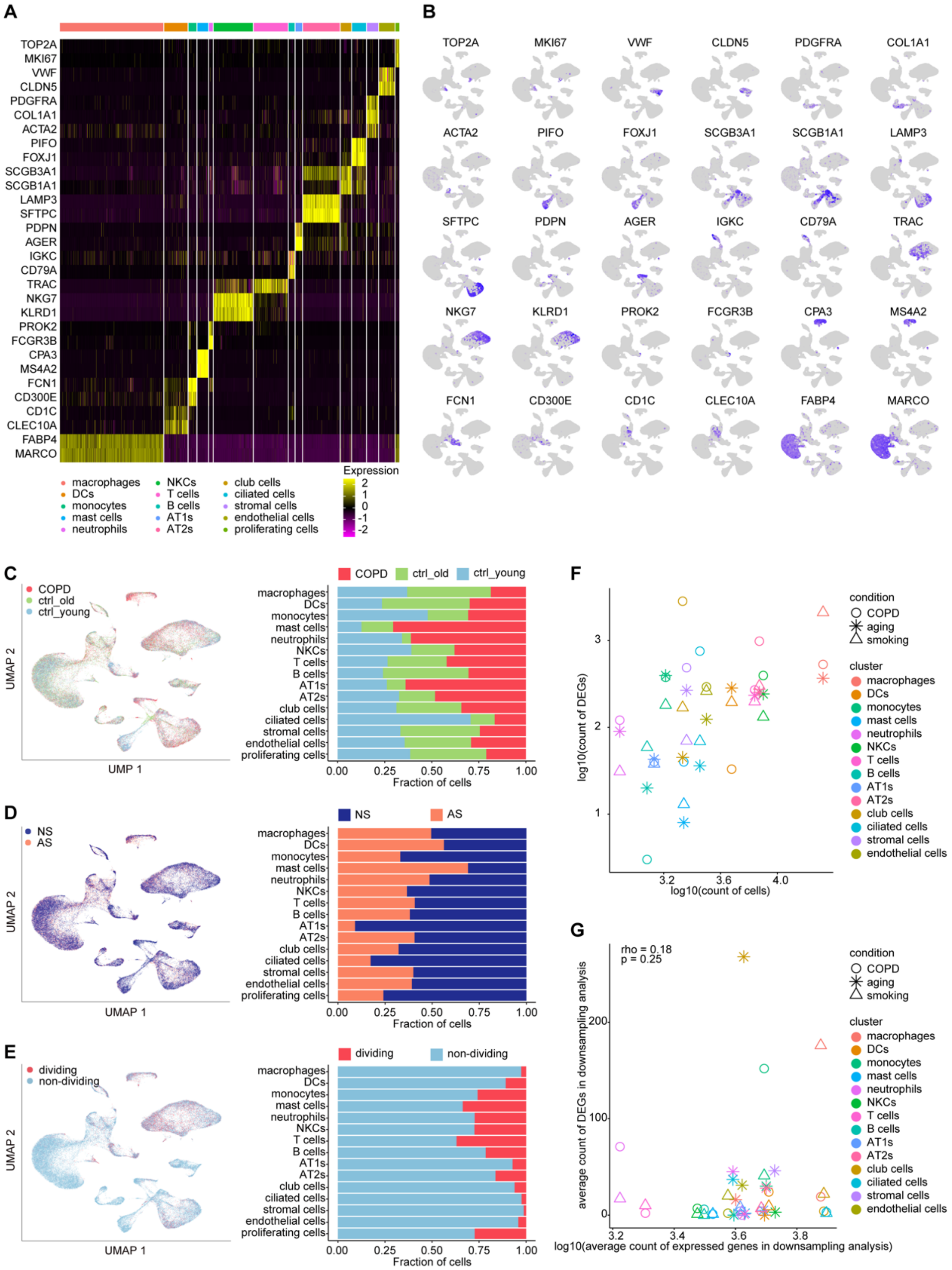

A
TOP2A
MKI67
VWF
CLDN5
PDGFRA
COL1A1
ACTA2
PIFO
FOXJ1
SCGB3A1
SCGB1A1
LAMP3
SFTPC
PDPN
AGER
IGKC
CD79A
TRAC
NKG7
KLRD1
PROK2
FCGR3B
CPA3
MS4A2
FCN1
CD300E
CD1C
CLEC10A
FABP4
MARCO
Expression
2
1
0
-1
-2
macrophages
DCs
monocytes
mast cells
neutrophils
NKCs
T cells
B cells
AT1s
AT2s
club cells
ciliated cells
stromal cells
endothelial cells
proliferating cells
B
C
COPD
ctrl_old
ctrl_young
UMAP 2
UMP 1
Fraction of cells
0.00
0.25
0.50
0.75
1.00
D
NS
AS
UMAP 1
E
dividing
non-dividing
F
condition
COPD
aging
smoking
cluster
log10(count of DEGs)
log10(count of cells)
G
rho = 0.18
p = 0.25
average count of DEGs in downsampling analysis
log10(average count of expressed genes in downsampling analysis)

Figure S2.

(**A**) Heatmap showing the expression of marker genes in each cell type used for cluster annotation in Figure 2A.

(**B**) Feature expression of the marker genes on UMAP plot used for cluster annotation in Figure 2A.

(**C**) UMAP plot representation of 66,610 cells from lung tissue samples of 3 male COPD patients, 3 male age-matched controls, and 3 male young normal individual; each dot represents a single cell (left-panel). The fractions of each cell type under group are shown in the box plot (right-panel).

(**D**) UMAP plot representation of 66,610 cells from lung tissue samples of 4 active smokers (AS) and 5 never-smokers (NS); each dot represents a single cell (left-panel). The fractions of each cell type under smoking status are shown in the box plot (right-panel).

(**E**) UMAP plot representation of 66,610 cells including 9,508 dividing cell states and 57,102 non-dividing cell states; each dot represents a single cell (left-panel). The fractions of each cell type under cell dividing state are shown in the box plot (right-panel).

(**F**) Scatter plot showing the numbers of cell-type specific DEGs under different conditions (COPD, aging, and smoking) against the sample size (cell count) of the same cell type.

(**G**) Scatter plot showing the numbers of cell-type specific DEGs in downsampling analysis under different conditions (COPD, aging, and smoking) against the count of expressed genes of the same cell type in downsampling analysis. The count of cell-type specific DEGs is not dependent on the count of expressed genes of the same cell type. P: spearman's rank correlation p-value between the average numbers of DEGs and the average number of genes expressed in a given cell type in downsampling analysis. two-sided paired t-tests. rho: spearman's rank correlation coefficient.

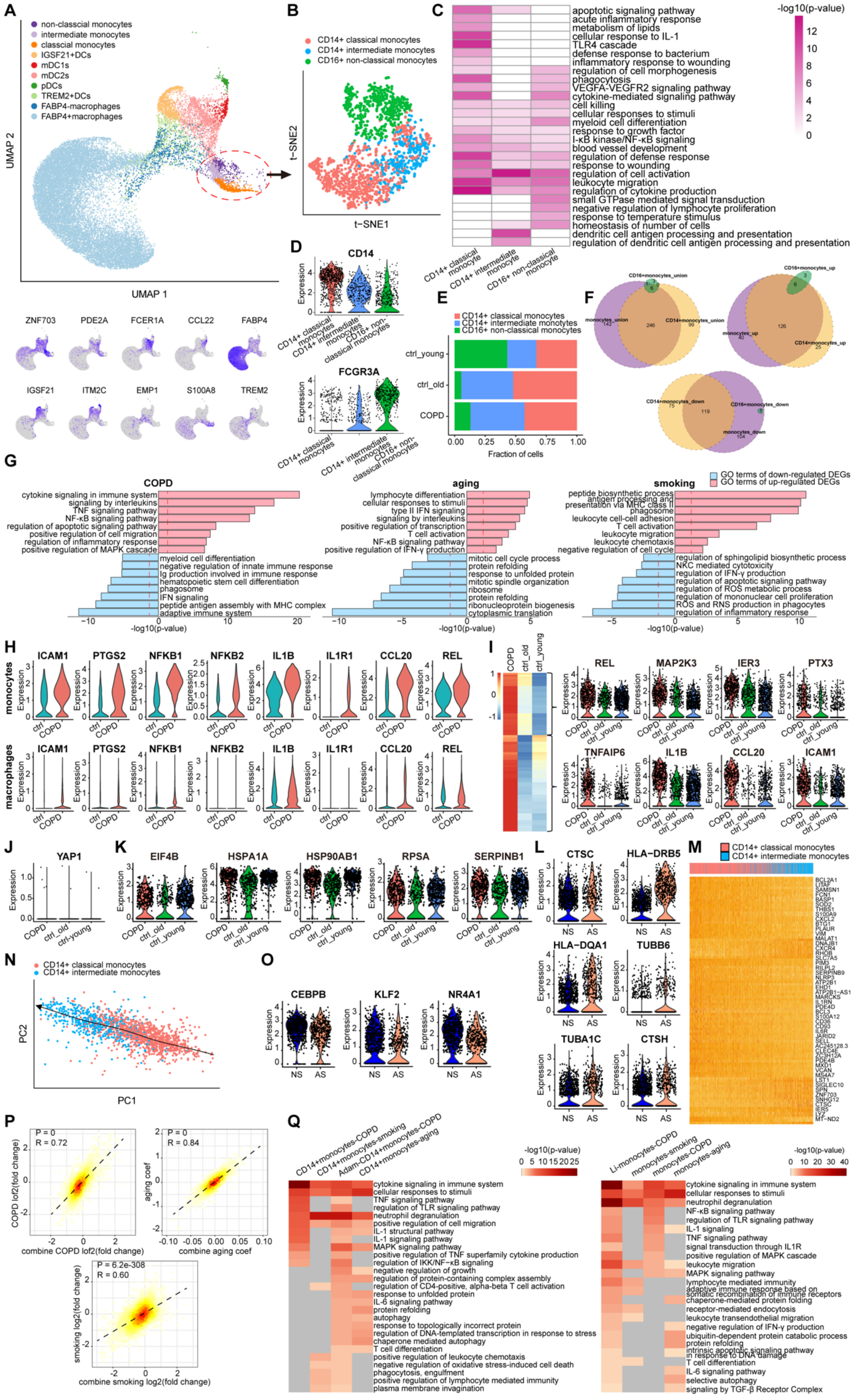

Figure S3.

(**A**) UMAP plot showing all the sub-clusters of monocytes, macrophages, and DCs, with each sub-cluster color-coded for annotated subtype, in which the part circled by the red dotted line are monocytes (up). Feature expression of the marker genes on UMAP plot used for sub-cluster annotation (bottom).

(**B**) t-SNE plot of 3 sub-clusters in monocytes, including CD14+ classical monocytes, CD14+ intermediate monocytes, and CD16+ non-classical monocytes. Cells are colored by sub-cluster type.

(**C**) Heatmap displaying the functional annotation of the 3 sub-clusters-specific expressed genes of monocytes in (**b**), highlighting the functional characteristic of each sub-cluster, such as the involvement of CD14+ classical monocytes in inflammation, immune response, as well as phagocytosis, and the ability of antigen presentation and T cell activation of CD14+ intermediate monocytes. The color of heatmap indicates high (dark pink) or low (white) -log10(p-value).

(**D**) Violin plots showing the expression of representative CD14+ classical monocytes marker gene (*CD14*) and CD16+ non-classical monocytes marker gene (*FCGR3A*/*CD16*) in 3 sub-clusters of monocytes.

(**E**) Bar plot showing the relative proportion of CD14+ classical monocytes, CD14+ intermediate monocytes, and CD16+ non-classical monocytes across young control (ctrl_young), old control (ctrl_old), and COPD patient (COPD) groups. Only CD16+ non-classical monocytes are primarily derived from young control group.

(**F**) Venn plots of total (union, up left), up-regulated (up, up right), and down-regulated (down, bottom) COPD-associated DEGs in total monocytes, CD14+ monocytes, and CD16+ monocytes, clearly showing the overrepresentation of all the 3 kinds of COPD-associated DEGs in CD14+ monocytes, compared with CD16+ monocytes.

(**G**) Bar plots showing the top GO functional enrichment of COPD- (left), aging- (middle), and smoking- (right) DEGs of CD14+ monocytes which are arranged according to p-value.

(**H**) Violin plots demonstrating the expression distributions of *ICAM1, PTGS2, NFKB1, NFKB2, IL1B, IL1R1, CCL20,* and *REL* in monocytes (up) and macrophages (bottom) among control and COPD groups, revealing the distinction in activation of these pro-inflammatory genes between monocytes and macrophages under COPD conditions.

(**I**) Heatmap displaying the average expression of the up-regulated COPD-associated DEGs in monocytes among three groups (COPD, ctrl_young, and ctrl_young) (left), from which upstream genes (such as *REL*, *MAP2K3*, *IER3*, and *PTX3*) in NF-κB and TNF pathways are also increased in monocytes from old controls (up right), while many downstream effectors (such as *TNFAIP6*, *IL1B*, *CCL20*, and *ICAM1*) are only marginally increased or even decreased in monocytes from old controls, compared with young controls (bottom right). The color of heatmap indicates high (red) or low (blue) expression value.

(**J**) Violin plot showing the expression of *YAP1* in monocytes among three groups (COPD, ctrl_young, and ctrl_young), suggesting that *YAP1* is hardly expressed in monocytes.

(**K**) Violin plots displaying the specific down-regulation of several aging-associated DEGs of monocytes including translation initiation factors (*EIF4B*), heat shock proteins (*HSP90AB1*, *HSPA1A*), ribosomal proteins (*RPSA*), and protease inhibitors vital to inflammation as well as cellular secretion (*SERPINB1*) in monocytes from old controls, compared with COPD patients and young controls, indicating the post-transcriptional dysfunction of aged monocytes accounting for inflammaging.

(**L**) Violin plots showing the expression distributions of some representative genes enriched in MHC class II antigen presentation (including *CTSC*, *HLA-DRB5*, *HLA-DQA1*, *TUBB6*, *TUBA1C*, and *CTSH*) in monocytes among active smoker (AS) and never-smoker (NS) groups, the increased expression of which in active smoker (AS) groups strongly indicates the activation of antigen processing and presentation via MHC II in the monocytes from active smokers (AS), compared with never-smokers (NS).

(**M**) Heatmaps displaying the scaled expression profile for the top 100 genes (largest waldStat from slingshot associationTest) contributing to CD14+ classical monocytes-to-CD14+ intermediate monocytes differentiation with cells (columns) ordered according to their pseudotimes.

(**N**) A trajectory reflecting CD14+ classical monocytes-to-CD14+ intermediate monocytes differentiation by slingshot.

(**O**) Violin plots displaying the expression of *CEBPB*, *KLF2*, and *NR4A1* in monocytes among active smoker (AS) and never-smoker (NS) groups, showing that these genes vital to monocyte differentiation are all declined by smoking.

(**P**) Scatter plots showing the highly positive correlation between the log2(fold change) of the COPD-, smoking-, as well as aging-associated DEGs of monocytes from our dataset and those combined with the correspondent data from Adams and his colleagues (32832599), respectively. P: Pearson correlation p-value of two-sided paired t-tests. R: Pearson correlation coefficient of two-sided paired t-tests.

(**Q**) Terms with the best p-value within the COPD-, aging-, and smoking-associated DEGs of monocytes in our dataset and those within the COPD-associated DEGs of monocytes in Li's (33823868) are selected to eliminate some biological processes too broad to reflect the specific pathological role and displayed in a heatmap (left). Terms with the best p-value within the COPD-, aging-, and smoking-associated DEGs of CD14+ monocytes in our dataset and those within the COPD-associated DEGs of CD14+ monocytes in Adams' (32832599) are also selected and displayed in a heatmap (right). Both heatmaps demonstrate similarities of the molecular changes in monocytes from COPD lung tissues between our dataset and others', while also highlighted the distinct molecular changes in monocytes from aged and smoking lung tissues detected in our dataset which are to some degree overlapped with those of monocytes from COPD lung tissues in others' datasets due to their mixture with aging and smoking.

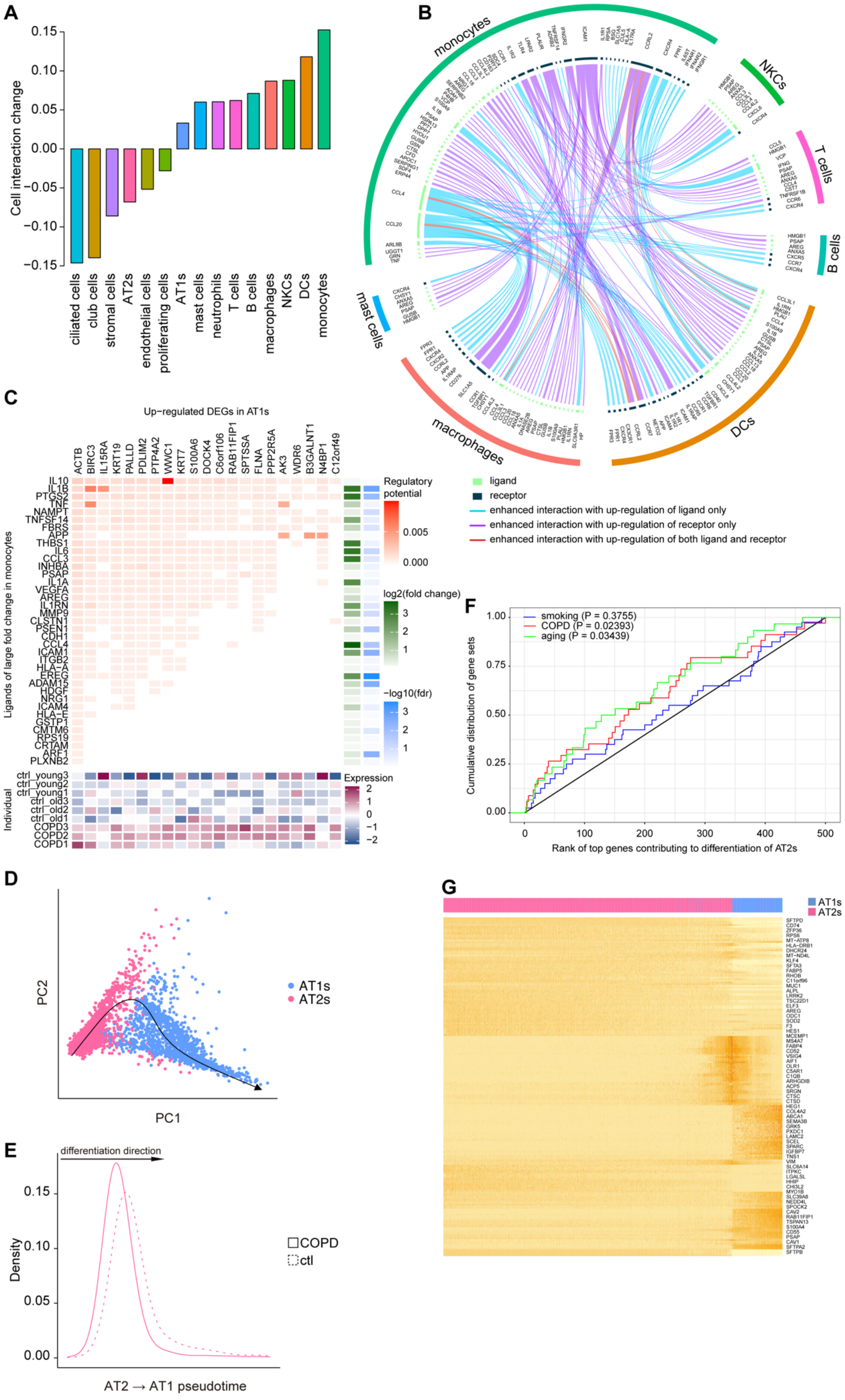
A
Cell interaction change
0.15
0.10
0.05
0.00
−0.05
−0.10
−0.15
ciliated cells
club cells
stromal cells
AT2s
endothelial cells
proliferating cells
AT1s
mast cells
neutrophils
T cells
B cells
macrophages
NKCs
DCs
monocytes
B
monocytes
NKCs
T cells
B cells
DCs
macrophages
mast cells
ligand
receptor
enhanced interaction with up-regulation of ligand only
enhanced interaction with up-regulation of receptor only
enhanced interaction with up-regulation of both ligand and receptor
C
Up−regulated DEGs in AT1s
ACTB BIRC3 IL15RA KRT19 PALLD PDLIM2 PTP4A2 WWC1 KRT7 S100A6 DOCK4 C6orf106 RAB11FIP1 SPTSSA FLNA PPP2R5A AK3 WDR6 B3GALNT1 N4BP1 C12orf49
Ligands of large fold change in monocytes
IL10 IL1B PTGS2 TNF NAMPT TNFSF14 FBRS APP THBS1 IL6 CCL3 INHBA PSAP IL1A VEGFA AREG IL1RN MMP9 CLSTN1 PSEN1 CDH1 CCL4 ICAM1 ITGB2 HLA−A EREG ADAM15 HDGF NRG1 ICAM4 HLA−E GSTP1 CMTM6 RPS19 CRTAM ARF1 PLXNB2
Regulatory potential
0.005
0.000
log2(fold change)
3
2
1
−log10(fdr)
3
2
1
Individual
ctrl_young3 ctrl_young2 ctrl_young1 ctrl_old3 ctrl_old2 ctrl_old1 COPD3 COPD2 COPD1
Expression
2
1
0
−1
−2
D
PC2
PC1
AT1s
AT2s
E
differentiation direction
Density
0.15
0.10
0.05
0.00
COPD
ctl
AT2 → AT1 pseudotime
F
smoking (P = 0.3755)
COPD (P = 0.02393)
aging (P = 0.03439)
Cumulative distribution of gene sets
1.00
0.75
0.50
0.25
0.00
0
100
200
300
400
500
Rank of top genes contributing to differentiation of AT2s
G
AT1s
AT2s

Figure S4.

(**A**) Bar plot showing the average relative changes of CCIs of each cell type with all the cell types predicted by CellPhoneDB from control lungs to COPD lungs. For example, in monocytes, the relative change (the difference between the COPD significant ligand-receptor (LR) interaction pair number and the control significant LR interaction pair number and then divided by the control significant LR interaction pair number) of CCI between monocytes and each cell types under the two conditions of COPD and control are calculated first, and then the mean of the relative change across all cell types are calculated as monocytes average relative changes of CCIs.

(**B**) Circos plot showing the ligand-receptor pairs responsible for the enhanced CCIs among monocytes, NKCs, T cells, B cells, DCs, macrophages, and mast cells predicted by CellPhoneDB, for which monocytes are either sender cells or receiver cells. Each light green inside arc represents a ligand and each dark green one represents a receptor. Blue lines link ligands and receptors showing enhanced interactions with up-regulation of ligands only. Purple lines link ligands and receptors showing enhanced interactions with up-regulation of receptors only. Red lines link ligands and receptors showing enhanced interactions with up-regulation of both ligands and receptors.

(**C**) The top heatmap showing the potential of ligands of large fold change (log2(fold change) > 0.14 or log2(fold change) < -0.14) in monocytes (on the y-axis) regulating the up-regulated COPD-associated DEGs in AT1s (on the x-axis) predicted by NicheNet analysis. The fold change and false discovery rate of these ligands are shown in 2 separate heatmaps on the right. The mean gene expressions of the up-regulated COPD-associated DEGs in AT1s across 9 samples are shown in the lower heatmap. The mean gene expression per sample is represented as a z-transformed value (across all samples).

(**D**) A trajectory reflecting AT2-to-AT1 differentiation by slingshot.

(**E**) Density plot of cells from control (dotted line) and COPD (full line) lungs in AT1s and AT2s along AT2s to AT1s pseudotime. Cells from COPD lung tissues are more clustered in the early stage of differentiation path, supporting compromised capacity of AT2s differentiating to AT1s during COPD development.

(**F**) Kolmogorov-Smirnov plot showing the enrichment of COPD-, aging-, and smoking-associated DEGs of monocytes among the set of top 500 driver genes (largest waldStat from slingshot associationTest) responsible for AT2-to-AT1 differentiation, highlighting the overrepresentation of both COPD- and aging-associated DEGs among the set of driver genes.

(**G**) Heatmaps displaying the scaled expression profile for the top 100 driver genes (largest waldStat from slingshot associationTest) contributing to AT2-to-AT1 differentiation with cells (columns) ordered according to their pseudotimes.

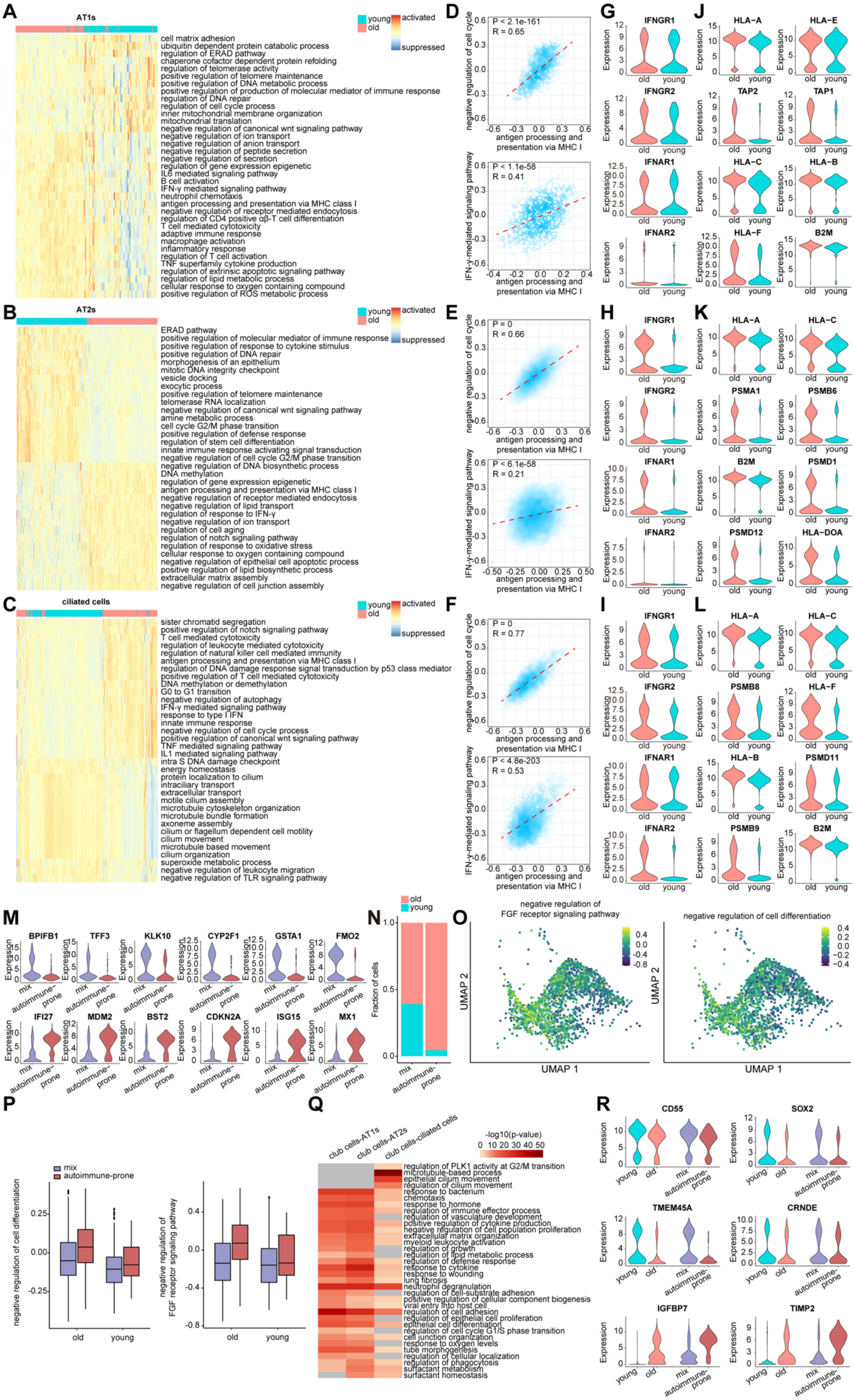
A
AT1s
young
old
activated
suppressed
cell matrix adhesion
ubiquitin dependent protein catabolic process
regulation of ERAD pathway
chaperone cofactor dependent protein refolding
regulation of telomerase activity
positive regulation of telomere maintenance
positive regulation of DNA metabolic process
positive regulation of production of molecular mediator of immune response
regulation of DNA repair
regulation of cell cycle process
inner mitochondrial membrane organization
mitochondrial translation
negative regulation of canonical wnt signaling pathway
negative regulation of ion transport
negative regulation of anion transport
negative regulation of peptide secretion
negative regulation of secretion
regulation of gene expression epigenetic
IL6 mediated signaling pathway
B cell activation
IFN-γ mediated signaling pathway
neutrophil chemotaxis
antigen processing and presentation via MHC class I
negative regulation of receptor mediated endocytosis
regulation of CD4 positive αβ-T cell differentiation
T cell mediated cytotoxicity
adaptive immune response
macrophage activation
inflammatory response
regulation of T cell activation
TNF superfamily cytokine production
regulation of extrinsic apoptotic signaling pathway
regulation of lipid metabolic process
cellular response to oxygen containing compound
positive regulation of ROS metabolic process
B
AT2s
ERAD pathway
positive regulation of molecular mediator of immune response
positive regulation of response to cytokine stimulus
positive regulation of DNA repair
morphogenesis of an epithelium
mitotic DNA integrity checkpoint
vesicle docking
exocytic process
positive regulation of telomere maintenance
telomerase RNA localization
negative regulation of canonical wnt signaling pathway
amine metabolic process
cell cycle G2/M phase transition
positive regulation of defense response
regulation of stem cell differentiation
innate immune response activating signal transduction
negative regulation of cell cycle G2/M phase transition
negative regulation of DNA biosynthetic process
DNA methylation
regulation of gene expression epigenetic
antigen processing and presentation via MHC class I
negative regulation of receptor mediated endocytosis
negative regulation of lipid transport
regulation of response to IFN-γ
negative regulation of ion transport
regulation of cell aging
regulation of notch signaling pathway
regulation of response to oxidative stress
cellular response to oxygen containing compound
negative regulation of epithelial cell apoptotic process
positive regulation of lipid biosynthetic process
extracellular matrix assembly
negative regulation of cell junction assembly
C
ciliated cells
sister chromatid segregation
positive regulation of notch signaling pathway
T cell mediated cytotoxicity
regulation of leukocyte mediated cytotoxicity
regulation of natural killer cell mediated immunity
antigen processing and presentation via MHC class I
regulation of DNA damage response signal transduction by p53 class mediator
positive regulation of T cell mediated cytotoxicity
DNA methylation or demethylation
G0 to G1 transition
negative regulation of autophagy
IFN-γ mediated signaling pathway
response to type I IFN
innate immune response
negative regulation of cell cycle process
positive regulation of canonical wnt signaling pathway
TNF mediated signaling pathway
IL1 mediated signaling pathway
intra S DNA damage checkpoint
energy homeostasis
protein localization to cilium
intraciliary transport
extracellular transport
motile cilium assembly
microtubule cytoskeleton organization
microtubule bundle formation
axoneme assembly
cilium or flagellum dependent cell motility
cilium movement
microtubule based movement
cilium organization
superoxide metabolic process
negative regulation of leukocyte migration
negative regulation of TLR signaling pathway
D
P < 2.1e-161
R = 0.65
negative regulation of cell cycle
antigen processing and presentation via MHC I
P < 1.1e-58
R = 0.41
IFN-γ-mediated signaling pathway
E
P = 0
R = 0.66
P < 6.1e-58
R = 0.21
F
P = 0
R = 0.77
P < 4.8e-203
R = 0.53
G
IFNGR1
IFNGR2
IFNAR1
IFNAR2
Expression
old
young
J
HLA-A
HLA-E
TAP2
TAP1
HLA-C
HLA-B
HLA-F
B2M
H
K
PSMA1
PSMB6
PSMD1
PSMD12
HLA-DOA
I
L
PSMB8
PSMB9
PSMD11
M
BPIFB1
TFF3
KLK10
CYP2F1
GSTA1
FMO2
IFI27
MDM2
BST2
CDKN2A
ISG15
MX1
mix
autoimmune-prone
N
Fraction of cells
O
negative regulation of FGF receptor signaling pathway
negative regulation of cell differentiation
UMAP 1
UMAP 2
P
negative regulation of cell differentiation
negative regulation of FGF receptor signaling pathway
Q
club cells-AT1s
club cells-AT2s
club cells-ciliated cells
-log10(p-value)
regulation of PLK1 activity at G2/M transition
microtubule-based process
epithelial cilium movement
regulation of cilium movement
response to bacterium
chemotaxis
response to hormone
regulation of immune effector process
regulation of vasculature development
positive regulation of cytokine production
negative regulation of cell population proliferation
extracellular matrix organization
myeloid leukocyte activation
regulation of growth
regulation of lipid metabolic process
regulation of defense response
response to cytokine
response to wounding
lung fibrosis
neutrophil degranulation
regulation of cell-substrate adhesion
positive regulation of cellular component biogenesis
viral entry into host cell
regulation of cell adhesion
regulation of epithelial cell proliferation
epithelial cell differentiation
regulation of cell cycle G1/S phase transition
cell junction organization
response to oxygen levels
tube morphogenesis
regulation of cellular localization
regulation of phagocytosis
surfactant metabolism
surfactant homeostasis
R
CD55
SOX2
TMEM45A
CRNDE
IGFBP7
TIMP2

Figure S5.

(**A**-**C**) Heatmaps showing the GSVA analysis enrichment score of the top altered biological pathways in AT1s (**A**), AT2s (**B**), and ciliated cells (**C**) from lung tissues of young (cyan) and old (pink) individuals. All cells within young or old are randomly clustered into 100 groups, and then averages of expression were calculated within each group, resulting in 100 transcriptomic profiles each for old and young. The colors from red to blue represent alterations of biological pathways states from activated to suppressed.

(**D**-**F**) Scatterplots showing the correlations of GSVA analysis enrichment score between pathway of antigen processing and presentation via MHC class I and two other biological pathways (including negative regulation of cell cycle and IFN-γ-mediated signalling pathway) in AT1s (**D**), AT2s (**E**), and ciliated cells (**F**) from lung tissues of young (cyan) and old (pink) individuals. P: Pearson correlation p-value of two-sided paired t-tests. R: Pearson correlation coefficient of two-sided paired t-tests.

(**G**-**I**) Violin plots for genes encoding IFN receptors including *IFNRG1*, *IFNRG2*, *IFNRA1*, and *IFNRA2* in AT1s (**G**), AT2s (**H**), and ciliated cells (**I**) from lung tissues of young (cyan) and old (pink) individuals, demonstrating the up-regulation of IFN receptors responsible for the generally enhanced IFN reactivity in aged lung epithelial cells.

(**J**-**L**) Violin plots for genes involved in pathway of antigen processing and presentation via MHC class I in AT1s (**J**), AT2s (**K**), and ciliated cells (**L**) from lung tissues of young (cyan) and old (pink) individuals, demonstrating the up-regulation of key components of MHC class I antigen presentation pathway (esp. *B2M*) responsible for the generally augmented autoimmunity in aged lung epithelial cells.

(**M**) Violin plots showing the expression levels of marker genes for the mix sub-cluster of club cells (*BPIFB1*, *TFF3*, *KLK10*, *CYP2F1*, *GSTA1*, and *FMO2*) and those for the autoimmune-prone sub-cluster of club cells (*IFI27*, *MDM2*, *BST2*, *CDKN2A*, *ISG15*, and *MX1*) in both sub-clusters of club cells.

(**N**) Bar plot showing percentages of club cells from lung tissues of young (cyan) and old (pink) individuals in autoimmune-prone sub-cluster of club cells (autoimmune-prone) and other sub-clusters of club cells (mix). The enrichment of autoimmune-prone sub-cluster in club cells from lung tissues of old individuals highlightes the autoimmunity unique to aged club cells.

(**O**) Feature plots for GSVA analysis enrichment scores of FGF receptor signalling pathway (left) and negative regulation of cell differentiation (right) in all the club cells. One dot represents a club cell. The colors from yellow to dark green represent alterations of biological pathways states from activated to suppressed. Both FGF

receptor signalling and cell differentiation are relatively suppressed in the autoimmune-prone sub-cluster of club cells.

(**P**) Box plots showing the GSVA analysis enrichment score of negative regulation of cell differentiation and negative regulation of FGF receptor signalling pathway in autoimmune-prone and mix sub-clusters among club cells from lung tissues of young and old individuals. Both pathways are significantly activated in the autoimmune-prone sub-cluster among club cells from lung tissues of young individuals, compared to the mix one among the club cells from lung tissues of the same individuals, the degree of which is much greater among the club cells from lung tissues of the old individuals.

(**Q**) Functional annotation of top 100 driver genes in the three differentiation paths of club cells, including club cells to AT1s, club cells to AT2s, and club cells to ciliated cells, which not only includes genes that are generally important to cell differentiation, but also highlights functional genes specific to different cell types. The heatmap is used to visualize these biological processes, of which cells are colored by their enrichment analysis p-values and the gray cells indicate the lack of enrichment for that term in the corresponding gene list.

(**R**) Violin plots for pro-proliferative genes (including *SOX2*, *TMEM45A*, *CRNDE*, and *CD55*) and anti-proliferative ones (including *IGFBP7* and *TIMP2*) expression in club cells from lung tissues of young (cyan) and old (pink) individuals as well as the autoimmune-prone (brown) and mix (purple) sub-clusters of club cells. The pro-proliferative genes are down-regulated in club cells from lung tissues of old individuals, compared with those from young ones. The anti-proliferative genes are just the opposite.

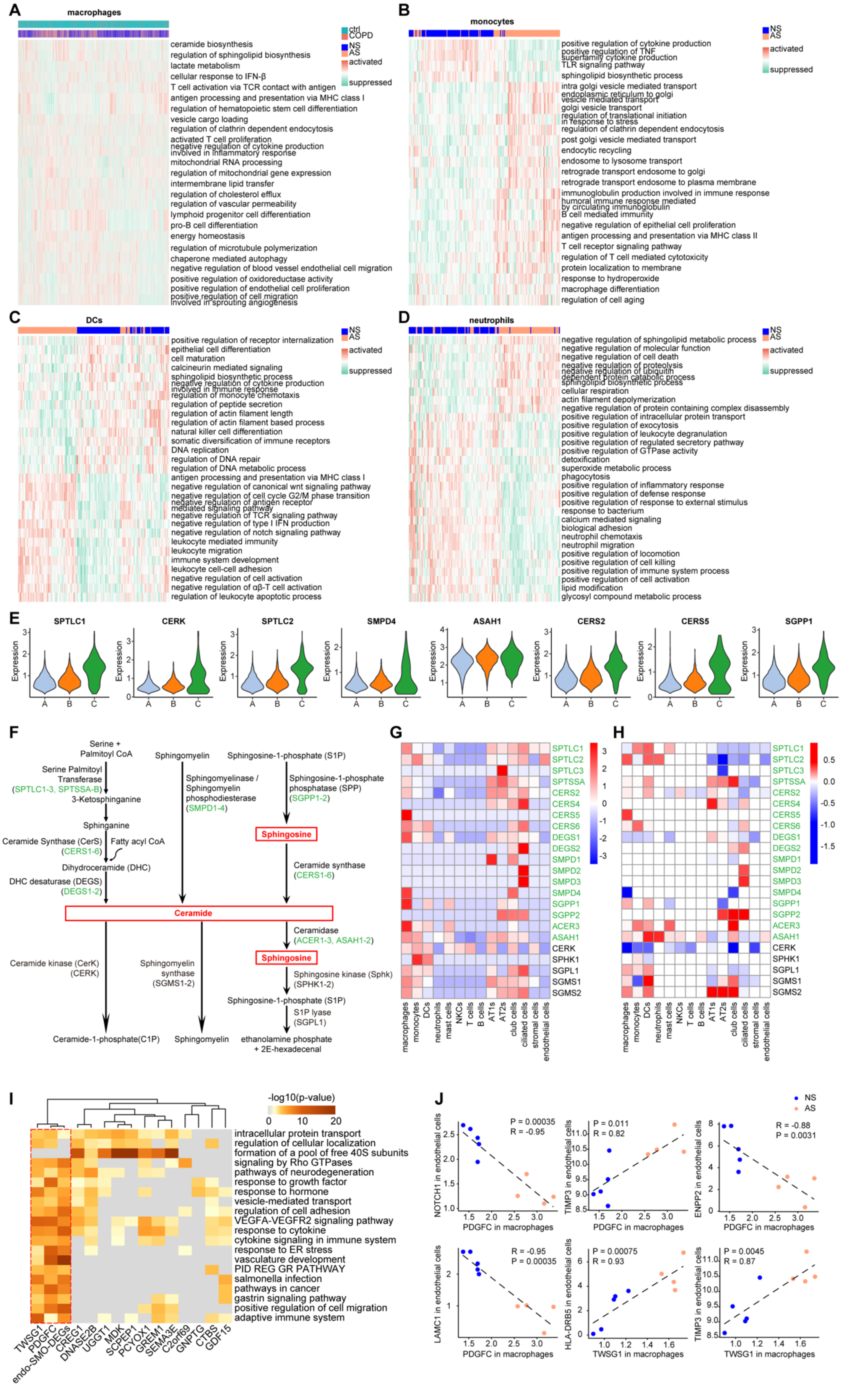
A
macrophages
ctrl
COPD
NS
AS
activated
suppressed
ceramide biosynthesis
regulation of sphingolipid biosynthesis
lactate metabolism
cellular response to IFN-β
T cell activation via TCR contact with antigen
antigen processing and presentation via MHC class I
regulation of hematopoietic stem cell differentiation
vesicle cargo loading
regulation of clathrin dependent endocytosis
activated T cell proliferation
negative regulation of cytokine production involved in inflammatory response
mitochondrial RNA processing
regulation of mitochondrial gene expression
intermembrane lipid transfer
regulation of cholesterol efflux
regulation of vascular permeability
lymphoid progenitor cell differentiation
pro-B cell differentiation
energy homeostasis
regulation of microtubule polymerization
chaperone mediated autophagy
negative regulation of blood vessel endothelial cell migration
positive regulation of oxidoreductase activity
positive regulation of endothelial cell proliferation
positive regulation of cell migration involved in sprouting angiogenesis
B
monocytes
positive regulation of cytokine production
positive regulation of TNF superfamily cytokine production
TLR signaling pathway
sphingolipid biosynthetic process
intra golgi vesicle mediated transport
endoplasmic reticulum to golgi vesicle mediated transport
golgi vesicle transport
regulation of translational initiation in response to stress
regulation of clathrin dependent endocytosis
post golgi vesicle mediated transport
endocytic recycling
endosome to lysosome transport
retrograde transport endosome to golgi
retrograde transport endosome to plasma membrane
immunoglobulin production involved in immune response
humoral immune response mediated by circulating immunoglobulin
B cell mediated immunity
negative regulation of epithelial cell proliferation
antigen processing and presentation via MHC class II
T cell receptor signaling pathway
regulation of T cell mediated cytotoxicity
protein localization to membrane
response to hydroperoxide
macrophage differentiation
regulation of cell aging
C
DCs
positive regulation of receptor internalization
epithelial cell differentiation
cell maturation
calcineurin mediated signaling
sphingolipid biosynthetic process
negative regulation of cytokine production involved in immune response
regulation of monocyte chemotaxis
regulation of peptide secretion
regulation of actin filament length
regulation of actin filament based process
natural killer cell differentiation
somatic diversification of immune receptors
DNA replication
regulation of DNA repair
regulation of DNA metabolic process
antigen processing and presentation via MHC class I
negative regulation of canonical wnt signaling pathway
negative regulation of cell cycle G2/M phase transition
negative regulation of antigen receptor mediated signaling pathway
negative regulation of TCR signaling pathway
negative regulation of type I IFN production
negative regulation of notch signaling pathway
leukocyte mediated immunity
leukocyte migration
immune system development
leukocyte cell-cell adhesion
negative regulation of cell activation
negative regulation of αβ-T cell activation
regulation of leukocyte apoptotic process
D
neutrophils
negative regulation of sphingolipid metabolic process
negative regulation of molecular function
negative regulation of cell death
negative regulation of proteolysis
negative regulation of ubiquitin dependent protein catabolic process
sphingolipid biosynthetic process
cellular respiration
actin filament depolymerization
negative regulation of protein containing complex disassembly
positive regulation of intracellular protein transport
positive regulation of exocytosis
positive regulation of leukocyte degranulation
positive regulation of regulated secretory pathway
positive regulation of GTPase activity
detoxification
superoxide metabolic process
phagocytosis
positive regulation of inflammatory response
positive regulation of defense response
positive regulation of response to external stimulus
response to bacterium
calcium mediated signaling
biological adhesion
neutrophil chemotaxis
neutrophil migration
positive regulation of locomotion
positive regulation of cell killing
positive regulation of immune system process
positive regulation of cell activation
lipid modification
glycosyl compound metabolic process
E
SPTLC1
CERK
SPTLC2
SMPD4
ASAH1
CERS2
CERS5
SGPP1
Expression
A
B
C
F
Serine + Palmitoyl CoA
Serine Palmitoyl Transferase (SPTLC1-3, SPTSSA-B)
3-Ketosphinganine
Sphinganine
Ceramide Synthase (CerS) (CERS1-6)
Fatty acyl CoA
Dihydroceramide (DHC)
DHC desaturase (DEGS) (DEGS1-2)
Sphingomyelin
Sphingomyelinase / Sphingomyelin phosphodiesterase (SMPD1-4)
Sphingosine-1-phosphate (S1P)
Sphingosine-1-phosphate phosphatase (SPP) (SGPP1-2)
Sphingosine
Ceramide synthase (CERS1-6)
Ceramide
Ceramidase (ACER1-3, ASAH1-2)
Sphingosine
Ceramide kinase (CerK) (CERK)
Sphingomyelin synthase (SGMS1-2)
Sphingosine kinase (Sphk) (SPHK1-2)
Sphingosine-1-phosphate (S1P)
S1P lyase (SGPL1)
Ceramide-1-phosphate(C1P)
Sphingomyelin
ethanolamine phosphate + 2E-hexadecenal
G
H
SPTLC1
SPTLC2
SPTLC3
SPTSSA
CERS2
CERS4
CERS5
CERS6
DEGS1
DEGS2
SMPD1
SMPD2
SMPD3
SMPD4
SGPP1
SGPP2
ACER3
ASAH1
CERK
SPHK1
SGPL1
SGMS1
SGMS2
macrophages
monocytes
DCs
neutrophils
mast cells
NKCs
T cells
B cells
AT1s
AT2s
club cells
ciliated cells
stromal cells
endothelial cells
I
-log10(p-value)
0
10
20
intracellular protein transport
regulation of cellular localization
formation of a pool of free 40S subunits
signaling by Rho GTPases
pathways of neurodegeneration
response to growth factor
response to hormone
vesicle-mediated transport
regulation of cell adhesion
VEGFA-VEGFR2 signaling pathway
response to cytokine
cytokine signaling in immune system
response to ER stress
vasculature development
PID REG GR PATHWAY
salmonella infection
pathways in cancer
gastrin signaling pathway
positive regulation of cell migration
adaptive immune system
TWSG1
PDGFC
endo-SMO-DEGs
CREG1
DNASE2B
UGGT1
MDK
SCPEP1
PCYOX1
GREM1
SEMA3E
C2orf69
GNPTG
CTBS
GDF15
J
NS
AS
P = 0.00035
R = -0.95
NOTCH1 in endothelial cells
PDGFC in macrophages
P = 0.011
R = 0.82
TIMP3 in endothelial cells
R = -0.88
P = 0.0031
ENPP2 in endothelial cells
R = -0.95
P = 0.00035
LAMC1 in endothelial cells
P = 0.00075
R = 0.93
HLA-DRB5 in endothelial cells
TWSG1 in macrophages
P = 0.0045
R = 0.87
TIMP3 in endothelial cells

Figure S6.

(**A**-**D**) Heatmaps showing the GSVA analysis enrichment score of the top altered biological pathways in macrophages (**A**), monocytes (**B**), DCs (**C**), and neutrophiles (**D**) from lung tissues of active smokers (AS, orange) and never-smokers (NS, blue). The colors from red to green represent alterations of biological pathways states from activated to suppressed. The disease conditions of individuals from which macrophages are derived are reflected as different colors of the bar above the heatmap in (**A**). The discrete distribution of macrophages in individual disease states reflectes their high heterogeneity.

(**E**) Violin plots for the expression of 8 genes encoding enzymes implicated in sphingolipid metabolism in subtypes A, B, and C of macrophages, showing the overexpression of *SPTLC1*, *SPTLC2*, *CERS2*, *CERS5*, *ASAH1*, *SGPP1*, *CERK*, and *SMPD4* in subtype C of macrophages.

(**F**) Schematic of sphingolipid metabolism. Highlighted in green are genes encoding the enzymes responsible for the biosynthesis of ceramide and sphingosine which are framed by red rectangular boxes.

(**G**) Heatmap showing the normalized average expression levels of genes encoding enzymes implicated in sphingolipid metabolism across all the cell types. The colors

from red to blue represent the gene expression levels from high to low. White represents no expression. Highlighted in green are genes encoding the enzymes responsible for the biosynthesis of ceramide and sphingosine. Only macrophages and airway epithelial cells (including AT1s, AT2s, club cells, and ciliated cells) demonstrate relatively high expressions of genes encoding the enzymes involved in the sphingolipid metabolic pathways.

(**H**) Heatmap showing the log2(fold change) of expression levels of genes encoding enzymes implicated in sphingolipid metabolism under smoking condition in the active smokers compared to the never-smokers across all the cell types. The colors from red to blue represent the gene expression levels from up-regulated to down-regulated. White represents genes no significant changes of expression or the gene expression value was too low to detect log2(fold change). Highlighted in green are genes encoding the enzymes responsible for the biosynthesis of ceramide and sphingosine. The up-regulation of several genes encoding enzymes catalyzing synthesis of ceramides (such as *SPTLC2*, *CERS2*, and *CERS5*) and sphingosines (such as *ASAH1* and *ACER3*) together with the down-regulation of genes encoding enzymes catalyzing catabolism of ceramides (such as *CERK*) in macrophages from smoking lungs support the elevated levels of ceramides and sphingosines in macrophages by smoking. In contrast, the expression of genes encoding enzymes involved in sphingolipids metabolism in endothelial cells is neither high in steady state nor significantly

changed under smoking conditions, ruling out the possibility that endothelial cells synthesize and secrete sphingolipids under smoking conditions.

(**I**) Functional annotation of smoking-associated DEGs in endothelial cells (endo-SMO-DEGs) and genes in endothelial cells highly correlated with up-regulated candidate ligands in macrophages analyzed in Fig. 7F (each list was named after the candidate ligand). The heatmap is used to visualize these biological processes, of which cells are colored by their enrichment analysis p-values and the gray cells indicate the lack of enrichment for that term in the corresponding gene list. Genes in endothelial cells correlated with ligand genes *PDGFC* and TWSG1 expressed in macrophages show the most overlap with smoking-associated DEGs in endothelial cells in terms of functional annotation, which is framed by a red dot rectangular box.

(**J**) Scatter plots showing cell type-specific mean gene expression within each individual correlations between *PDGFC* or *TWSG1* in macrophages and several representative highly correlated genes from endothelial cells across active smokers (AS) and never-smokers (NS).
